# Positive Selection and Multiple Losses of the LINE-1-Derived *L1TD1* Gene in Mammals Suggest a Dual Role in Genome Defense and Pluripotency

**DOI:** 10.1371/journal.pgen.1004531

**Published:** 2014-09-11

**Authors:** Richard N. McLaughlin, Janet M. Young, Lei Yang, Rafik Neme, Holly A. Wichman, Harmit S. Malik

**Affiliations:** 1 Division of Basic Sciences, Fred Hutchinson Cancer Research Center, Seattle, Washington, United States of America; 2 Department of Biological Sciences & Institute for Bioinformatics and Evolutionary Studies, University of Idaho, Moscow, Idaho, United States of America; 3 Max-Planck Institute for Evolutionary Biology, Plön, Germany; 4 Howard Hughes Medical Institute, Fred Hutchinson Cancer Research Center, Seattle, Washington, United States of America; BC Cancer Agency, Canada

## Abstract

Mammalian genomes comprise many active and fossilized retroelements. The obligate requirement for retroelement integration affords host genomes an opportunity to ‘domesticate’ retroelement genes for their own purpose, leading to important innovations in genome defense and placentation. While many such exaptations involve retroviruses, the *L1TD1* gene is the only known domesticated gene whose protein-coding sequence is almost entirely derived from a LINE-1 (L1) retroelement. Human *L1TD1* has been shown to play an important role in pluripotency maintenance. To investigate how this role was acquired, we traced the origin and evolution of *L1TD1*. We find that *L1TD1* originated in the common ancestor of eutherian mammals, but was lost or pseudogenized multiple times during mammalian evolution. We also find that *L1TD1* has evolved under positive selection during primate and mouse evolution, and that one prosimian *L1TD1* has ‘replenished’ itself with a more recent L1 ORF1 from the prosimian genome. These data suggest that *L1TD1* has been recurrently selected for functional novelty, perhaps for a role in genome defense. *L1TD1* loss is associated with L1 extinction in several megabat lineages, but not in sigmodontine rodents. We hypothesize that *L1TD1* could have originally evolved for genome defense against L1 elements. Later, *L1TD1* may have become incorporated into pluripotency maintenance in some lineages. Our study highlights the role of retroelement gene domestication in fundamental aspects of mammalian biology, and that such domesticated genes can adopt different functions in different lineages.

## Author Summary

Transposable elements comprise major portions of most animal genomes and are selfish genetic elements that may encode proteins needed for their own spread to new genomic locations. Though often considered genomic parasites, these elements also occasionally create novel genes that prove beneficial to the host, a process called 'domestication'. Here, we describe the evolution of a gene, *L1TD1*, which is derived from the protein-coding regions of the L1 mobile element family. We show that *L1TD1* was born in the common ancestor of placental mammals. *L1TD1* expression in stem cells and its requirement to maintain the pluripotent state of human embryonic stem cells suggested it might have been originally domesticated for such a pluripotency role. We find that *L1TD1*'s evolution does not fit with the predictions of this model; in fact, *L1TD1* has rapidly evolved in primates and mice and has been lost several times in mammals. We suggest an alternate model that *L1TD1* was born as a means to defend genomes against transposable elements, perhaps L1 itself. We propose that following this initial domestication, *L1TD1* later became incorporated into pluripotency programs in some mammalian lineages.

## Introduction

Retroelements have profoundly shaped mammalian genomes over millions of years. Insertion of these selfish elements can lead to gene inactivation or changes in transcriptional profiles of neighboring genes. Moreover, the presence of large stretches of almost identical sequence distributed across the genome poses recombinational hazards, leading to chromosomal rearrangements, often with pathological consequences. With a few celebrated exceptions [1], the retrotransposition activity of retroelements is rarely beneficial [2]. However, individual transposition events can occasionally drive dramatic episodes of adaptation through the generation of genetic novelty [3]. Indeed, repetitive elements have often been co-opted as transcriptional regulatory elements like promoters, enhancers, and insulators [4].

One particularly striking class of such genetic novelty results from the ‘domestication’ or ‘capture' of retroelement- and retrovirus-derived protein-coding genes by host genomes, a process by which these domesticated coding regions are exapted for a new function that is beneficial to the host [5], [6]. Such instances are often recognized by the preservation of individual protein-coding frames despite the mutational decay of the rest of the parental retroelement. This extinction of the replication-competent retroelement but not its protein-coding gene implies that selection for the benefit of the host genome must have prevented the mutational attrition of that particular gene.

Retroelements and retroviruses must integrate into the host genome as part of their replication cycle, presenting the host with a source of potentially advantageous protein-coding sequences. The *syncytin* genes of eutherian mammals represent some of the best characterized examples of such domestication events in which *envelope* genes from ancient retroviruses have been preserved for their membrane-fusing and/or immunosuppressive activities in the syncytiotrophoblast, the layer of the placenta which mediates maternal-fetal nutritional transfer [7]–[9]. In fact, loss of *syncytin-A* in mice is embryonic lethal, consistent with its indispensable role in placental function [10]. *Syncytin* genes thus represent a dramatic example of the maintenance and possibly invention of an essential function, placentation, via retroviral gene domestication. Importantly, *syncytin* is not unique in this regard; similar domestications of *sushi-ichi* LTR retrotransposon protein-coding regions have also created multiple host genes (e.g., *Peg10*) involved in genomic imprinting and placentation [11], [12].

Though *syncytin* represents a dramatic and beneficial genetic innovation, not all domesticated retroviral genes serve conserved, essential functions. The *Fv1* gene in mice represents a domesticated *gag* (viral capsid and nucleocapsid encoding) gene, which can actively interfere with the uncoating of incoming retroviral capsids [13], [14]. As expected for this genome defense role, *Fv1* is under strong diversifying selection, presumably as a result of constant innovation required to recognize and block different retroviruses [15]. As a result of this diversifying selection to chase the sequence of incoming capsids, orthologous genes can have dramatically different antiviral specificities [16]–[19]. *Fv1* has also been lost or pseudogenized at least twice in the *Mus* genus [15], perhaps because retention of *Fv1* may depend on persistence of selection from incoming viruses.

Mammalian genomes acquired both *syncytin* and *Fv1* genes as a result of the insertion of a retrovirus into the germline. Though such endogenous retroviruses have markedly impacted the mammalian genome, these elements have spent relatively little time coevolving with their host compared to the non-LTR retroposons, which date back at least to the origin of Metazoa [20]. The LINE-1 (Long INterspersed Element-1, L1) non-LTR retroposons make up a significant fraction of the human genome. Given their ancient history of coevolution with mammalian genomes, it is not surprising that there are numerous examples of exaptation of non-LTR retroelements into non-coding RNAs, as promoter or other regulatory elements, or as small portions of a coding region [4], . Many coding region exaptations are "exonizations", where cryptic splice sites within an intronic repetitive element are utilized so that a portion of the retroposon is incorporated as a novel exon of a host gene transcript, often as a minor splice isoform of the gene [25]–[28]. For example, a portion of an RTE non-LTR element was exapted as an additional coding exon of the existing ruminant *bucentaur* gene [29]. In contrast, only a single instance of a novel protein coding gene formed entirely anew from the domestication of a non-LTR retroposon has been described – *L1TD1*, or LINE-1 type Transposase Domain-containing 1 (L1TD1 has no known enzymatic activity despite its designation as a ‘transposase’ domain, see below). Though originally identified among a set of genes specifically expressed in murine embryonic stem cells, hence its original name *ECAT11* (Embryonic Stem Cell Associated Transcript 11) [30], *L1TD1's* origins and evolution remain poorly characterized.

In both human and mouse, *L1TD1* expression is high in undifferentiated stem cells and decreases precipitously upon differentiation [31]. In humans, L1TD1 is intricately linked with the cellular program for pluripotency maintenance, where it functions in a regulatory network with the core stem factors NANOG, SOX2, and OCT4. *L1TD1* expression appears to be tightly controlled by these pluripotency factors, which all bind the *L1TD1* promoter [32]. Depletion of *L1TD1* in human ES cells results in an immediate downregulation of *OCT4* (also known as *POU5F1*) and *NANOG*, and is sufficient to induce differentiation. In contrast, *L1TD1* in mice is completely dispensable for the maintenance and induction of pluripotency [30], [33]. These studies suggest that despite similarities in stem-cell specific expression, the function of *L1TD1* in pluripotency maintenance may have changed since the common ancestor of rodents and primates.

We wished to understand how a retroelement-derived gene could become incorporated into such an important cellular process in mammalian cells. To this end, we traced the evolutionary origins, history, and selective pressures of the *L1TD1* gene in mammals. In contrast to the expectation that *L1TD1* would be an essential gene if it had been domesticated for its function in mammalian pluripotency, we find several cases where *L1TD1* has been lost as well as several cases of selection for genetic innovations in *L1TD1*. This leads us to propose an evolutionary transition model wherein *L1TD1* was first retained for a role in genome defense in stem cells and germline (similar to *Fv1*). Subsequently, *L1TD1* may have been exapted for an essential function (similar to *syncytin*) in pluripotency maintenance in a subset of eutherian mammals.

## Results

### *L1TD1* originated in the common ancestor of placental mammals

**Figure 1:**
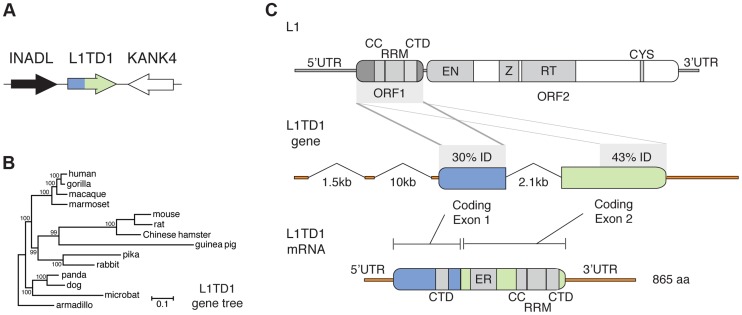
The mammalian *L1TD1* gene was born from a tandem insertion of L1 ORF1. A. Genomic context of *L1TD1*. Single-copy genes *INADL* and *KANK4* flank *L1TD1* in human, mouse and dog genomes; this shared syntenic arrangement helped us identify *L1TD1* orthologs in other mammalian genomes. B. *L1TD1* evolved according to the accepted species tree. We aligned *L1TD1* nucleotide sequences and generated a maximum-likelihood phylogeny (see Materials and Methods). Bootstrap values show the percentage of 1000 replicates in which descendent taxa cluster together, and the scale bar shows substitutions per site according to the GTR+I+G evolutionary model. C. *L1TD1* comprises two L1-ORF1p-like regions. L1 elements encode an approximately 6.5 kb transcript containing two open reading frames. L1 ORF1 encodes a protein, ORF1p, with RNA-binding and chaperone activity. ORF1p contains a coiled-coil motif (CC), a RNA-recognition motif (RRM), and a C-terminal domain (CTD). ORF2 encodes a protein with endonuclease and reverse transcription enzymatic functions. Sequence identity demonstrates that *L1TD1* was formed from the domestication of two copies of ORF1 from L1. The two copies may derive from independent insertions or from duplication after a single insertion. Human coding exon 1 and coding exon 2 share 30% and 43% amino acid identity, respectively, with ORF1p of human L1.3. In coding exon 1, only the CTD is conserved, while in coding exon 2, the CC, RRM, and CTD are all conserved. Coding exon 2 also contains a variable length glutamic acid-rich region (ER). After splicing, the human *L1TD1* transcript is 3849 nucleotides in length and encodes a single 865 amino acid protein product.

To gain insight into the cellular function of *L1TD1*, we decided to date its evolutionary origin and examine how its gene structure has changed since its birth. Since the only published analysis examined five mammalian genomes for its presence [33], we decided to search for the *L1TD1* gene in a much larger sample of diverse mammalian genomes. In the human and mouse genomes, *L1TD1* is found between the single-copy *INADL* and *KANK4* genes (Figure 1A). We found that all mammals and even bird genomes encode *INADL* and *KANK4* in close proximity to each other. We therefore used these flanking genes to identify the syntenic locus in other mammalian genomes, and used the sequence from this locus to identify *L1TD1* coding sequences where present. We also extended our search to the rest of the genome, using repeat-masked and unmasked human and mouse *L1TD1* sequences as BLAST search queries. We were never able to identify an intact copy of *L1TD1* outside the *INADL-KANK4* syntenic locus, although we did find some pseudogenes with obvious inactivating mutations in other locations. Although the previously published analysis [33] suggested that dog *L1TD1* lacks a portion of coding exon 1, in fact we find that *L1TD1* is complete and intact in the dog genome (Table S2).

We found *L1TD1* in many diverse placental mammalian genomes (Table S2, Dataset S1). However, *L1TD1* is absent from both marsupial and platypus genomes. This species distribution implies that the *L1TD1* gene was born in the common ancestor of placental mammals, after the split from marsupials. A phylogenetic tree using *L1TD1* nucleotide sequences follows the expected species tree (Figure 1B). Together with the observed shared syntenic location, this tree demonstrates that the sequences we identified represent truly orthologous genes rather than several independent L1 domestication events. This dates the origin of *L1TD1* to at least 106 million years ago, making it more ancient than the well-known domesticated retroelement genes *syncytin* and *Fv1* [9], [15].

### Two L1 ORF1-homologous regions constitute the *L1TD1* gene

Human *L1TD1* (RefSeq NM_001164835.1) comprises two protein-coding exons (of four total exons), which together encode an 865 amino acid protein. Each of these two exons is homologous to the first open reading frame (*ORF1*) of L1 (Figure 1C), whose protein product (ORF1p) functions as an RNA-binding protein that greatly enhances L1 retrotransposition [34]. ORF1p appears to be important in ensuring L1 ‘cis-preference’ - the preference for the ORF2 protein (ORF2p) to act upon the same RNA from which it was translated, so that an L1's machinery is less often ‘hijacked’ by other elements or other L1s [34]–[36]. *L1TD1* has no discernible homology to the other open reading frame of L1, ORF2, which encodes the enzymatic activities of L1. The second coding exon of *L1TD1* has higher conservation with ORF1p, showing 43% amino acid identity (58% amino acid similarity) with human L1 (L1.3), and apparent preservation of the coiled-coil, RNA-recognition, and C-terminal motifs of L1-ORF1p (Figure 1C). In contrast, the first coding exon retains only 30% amino acid identity (36% similarity) with ORF1p (L1.3), preserving the C-terminal domain but not the coiled-coil or RNA-recognition motif. This greater sequence identity in the second coding exon could either reflect a more recent origin from L1-ORF1 or greater constraint to preserve ancestral L1-ORF1p like functions. Coding exon 2 also encodes a ∼300 amino acid low-complexity glutamic acid-rich region that separates the two ORF1p-like regions. The annotated mouse *L1TD1* gene (RefSeq NM_001081202.1) is similar to human *L1TD1* but includes an extra intron that removes ∼200 nucleotides encoding ∼70 amino acids of this low-complexity region. However, we find that the reading frame is maintained through this mouse intron, and EST data suggest that it is often retained in the mature mouse *L1TD1* transcript. Overall, we find that *L1TD1* has maintained conservation with ORF1p of L1 despite its independent evolution for tens of millions of years; this suggests some aspect of L1 ORF1p function may still be utilized in L1TD1.

**Figure 2:**
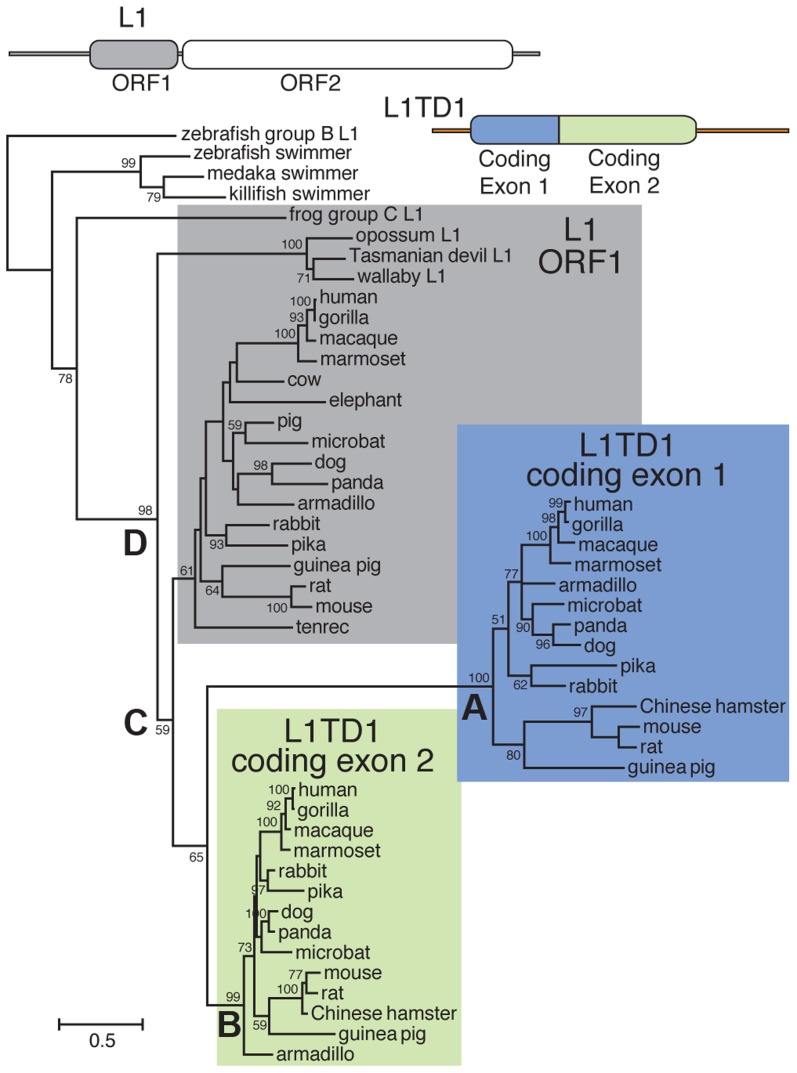
Phylogenetic tree of representative L1-ORF1p sequences and mammalian orthologs of the two *L1TD1* ORF1p-like regions. Predicted protein sequences of L1 ORF1p and the ORF1p-like regions encoded by each *L1TD1* exon were aligned and this alignment was used to generate a maximum-likelihood tree (see Materials and Methods). Bootstrap values show percentage of 1000 replicate trees in which the descendent taxa clustered together (only values >50% shown). The scale shows the number of substitutions per site. The tree was rooted using fish swimmer elements as outgroups. The N-terminal L1TD1 ORF1p-like sequences cluster together with a high bootstrap value (node A), as do the C-terminal L1TD1 ORF1p-like sequences (node B), confirming that we have identified true *L1TD1* orthologs and that the double ORF1p structure arose just once since the divergence of placental mammals and has not been subject to gene conversion between the two exons since. The L1TD1 N-terminal and C-terminal clades branch off from placental mammal L1-ORF1p sequences (node C) after marsupial and placental mammal L1-ORF1p sequences diverged (node D). The tree does not help to distinguish whether L1 ORF1 was independently domesticated twice, or just once with a subsequent genomic tandem duplication. Extensive sequence divergence between paralogous ORF1p-like sequence means that deep nodes of the tree are poorly resolved. Nonetheless, the tree supports our model in which both *L1TD1* exons were born after marsupials and placental mammals diverged.

To discern the order of the L1 domestication events that led to the origin of *L1TD1* and to confirm our dating of its birth, we generated a phylogeny of the two L1TD1 ORF1p-like amino acid sequences together with representative L1 ORF1p sequences from diverse mammalian genomes (Figure 2). We find that L1TD1 N-terminal regions (the protein product of coding exon 1) group together with strong bootstrap values (node A), as do L1TD1 C-terminal regions (the protein product of coding exon 2) (node B), demonstrating that the double ORF1p structure arose just once since the divergence of placental mammals and has not been subject to gene conversion between the two exons since. Furthermore, the L1TD1 N-terminal and C-terminal clades branch off from placental mammal L1-ORF1p sequences (node C) after marsupial and placental mammal L1-ORF1p sequences diverged (node D). This supports our conclusion that *L1TD1* was born via L1 domestication in the ancestral eutherian mammal. Our phylogeny cannot distinguish whether the two homologous regions of *L1TD1* derived from two independent L1 insertions in proximity to each other, or whether a single L1 inserted and subsequently experienced tandem duplication. Examination of the relative splice acceptor positions in coding exons 1 and 2 also does not help to distinguish these two possibilities. Nevertheless, it does appear that both coding exons were born in close temporal and physical proximity to each other, giving rise to the ancestral *L1TD1* gene. Our analyses further show that this bipartite double-ORF1p organization of *L1TD1* has been conserved since its birth.

### Multiple, independent losses of *L1TD1* in mammals

**Figure 3:**
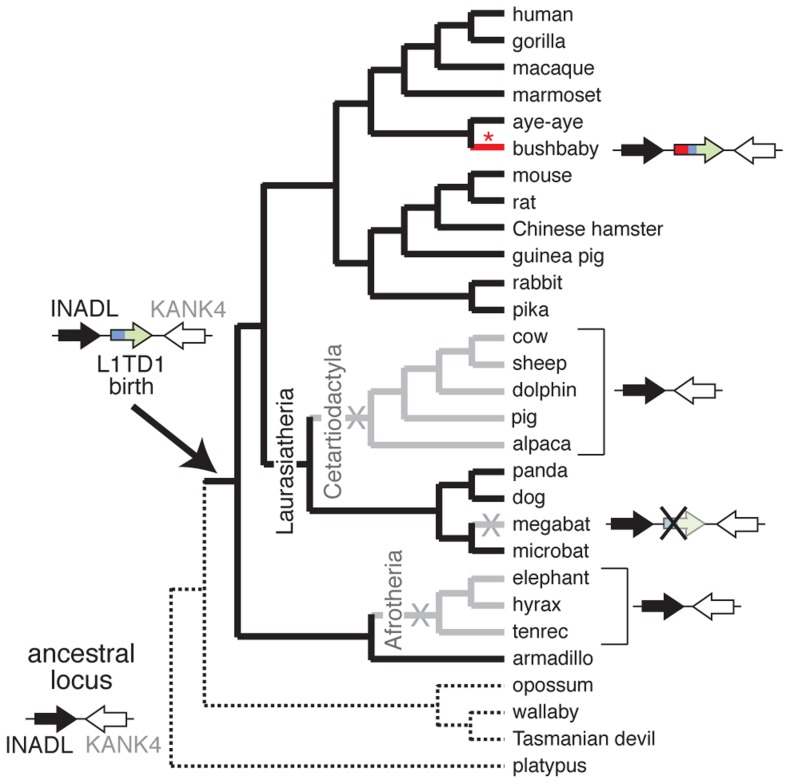
*L1TD1* has been lost multiple times in eutherian mammals. A species tree shows the presence or absence of *L1TD1* across mammals. Arrows depict *L1TD1*'s genomic locus; black (*INADL*) and white (*KANK4*) arrows depict the flanking genes we used to identify syntenic regions, and the blue/green arrow depicts *L1TD1*. *L1TD1*'s presence in the armadillo genome but not in platypus, opossum, wallaby or Tasmanian devil indicates it was most likely born before the divergence of placental mammals, but after divergence from marsupials (solid branches). *L1TD1* function was lost in three lineages (X's, and gray branches); it is present as a pseudogene in megabat and entirely missing from Afrotherian and Cetartiodactylan genomes. The bushbaby *L1TD1* gene acquired a novel N-terminal region (depicted in red) through a more recent L1 ORF1p domestication event (red asterisk) that occurred after bushbaby diverged from lemurs (Figure 4B).

Despite its widespread conservation, our genome-wide searches nevertheless revealed that *L1TD1* has been lost on at least three separate occasions during the evolution of placental mammals (Figure 3). First, we find that *L1TD1* is missing in all three Afrotherian genomes we surveyed – elephant, hyrax and tenrec. These three species constitute an approximately 85 million-year-old subclade. However, we find *L1TD1* is intact and present in the armadillo genome, which diverged from the three sequenced members of Afrotheria nearly 100 million years ago. Using parsimony, we infer that *L1TD1* was lost once in the ancestor of elephant, hyrax and tenrec between 84 and 100 million years ago.

Second, we find that *L1TD1* is missing from the genomes of all Cetartiodactyla. This clade of mammals originated 64 million years ago and comprises even-toed ungulates, whales, and dolphins. All sequenced genomes of this clade (cow, sheep, dolphin, pig and alpaca, Figure 3) lack *L1TD1*. In contrast to its loss in Cetartiodactyla, *L1TD1* is present in nearly all other members of the 80 million year old Laurasiatheria clade, which includes Cetartiodactyla. The most parsimonious explanation is that the common ancestor of Cetartiodactyla lost *L1TD1* between 64 and 80 million years ago.

In both the Afrotheria and Cetartiodactyla, we find no trace of the *L1TD1* gene anywhere in the genome, perhaps indicating loss of *L1TD1* via genomic deletions of the whole gene. However, given that the Afrotheria and Cetartiodactyla loss events likely occurred at least 64 and 84 million years ago, it is equally likely that after pseudogenization the *L1TD1* sequence has simply degenerated beyond recognition in these lineages.

The third instance of loss of *L1TD1* occurred in the genome of the megabat *Pteropus vampyrus* (Figure 3). The microbat *Myotis lucifugus* still encodes an intact *L1TD1*, but *P. vampyrus* contains a pseudogenized version of *L1TD1* with multiple frameshifts and stop codons. In contrast to the complete lack of a recognizable *L1TD1* in the Afrotheria and Cetartiodactyla, *L1TD1* is still discernible in the megabat, suggesting this loss may be more recent.

These three loss events in mammals strongly challenge the possibility that *L1TD1* was born into a role of pluripotency maintenance in the common ancestor of placental mammals, as we would expect such a gene to be essential and not subject to subsequent loss. It is formally possible that these lineages independently acquired another *L1TD1*-like activity that allowed the loss of the originally acquired *L1TD1*. However, it is more parsimonious that *L1TD1*'s role in pluripotency maintenance was acquired much later than its birth.

The loss of *L1TD1* in mammals parallels the multiple losses of the *Fv1* restriction factor in the *Mus* genus [15]. We therefore considered whether, like *Fv1*, *L1TD1* may have rapidly evolved under positive selection due to some role in a genetic conflict.

### Genetic innovation of primate and mouse *L1TD1* supports a ‘genetic conflict’ hypothesis

If *L1TD1* were indeed playing a role in genome defense against some pathogen, one hallmark of the ensuing conflict might be the rapid evolution of *L1TD1* coding sequence, a signature commonly seen at many host-virus interaction interfaces [37]. Such rapid change would be expected if the coevolving pathogen constantly evolved to evade *L1TD1* recognition, in which case *L1TD1* would be expected to rapidly evolve to ‘re-establish’ recognition of the rogue element. Indeed, it has been found that primate L1 ORF1p experienced an episode of adaptive evolution [38], consistent with the idea that L1 could be evolving to escape some sort of genome defense factor.

**Figure 4:**
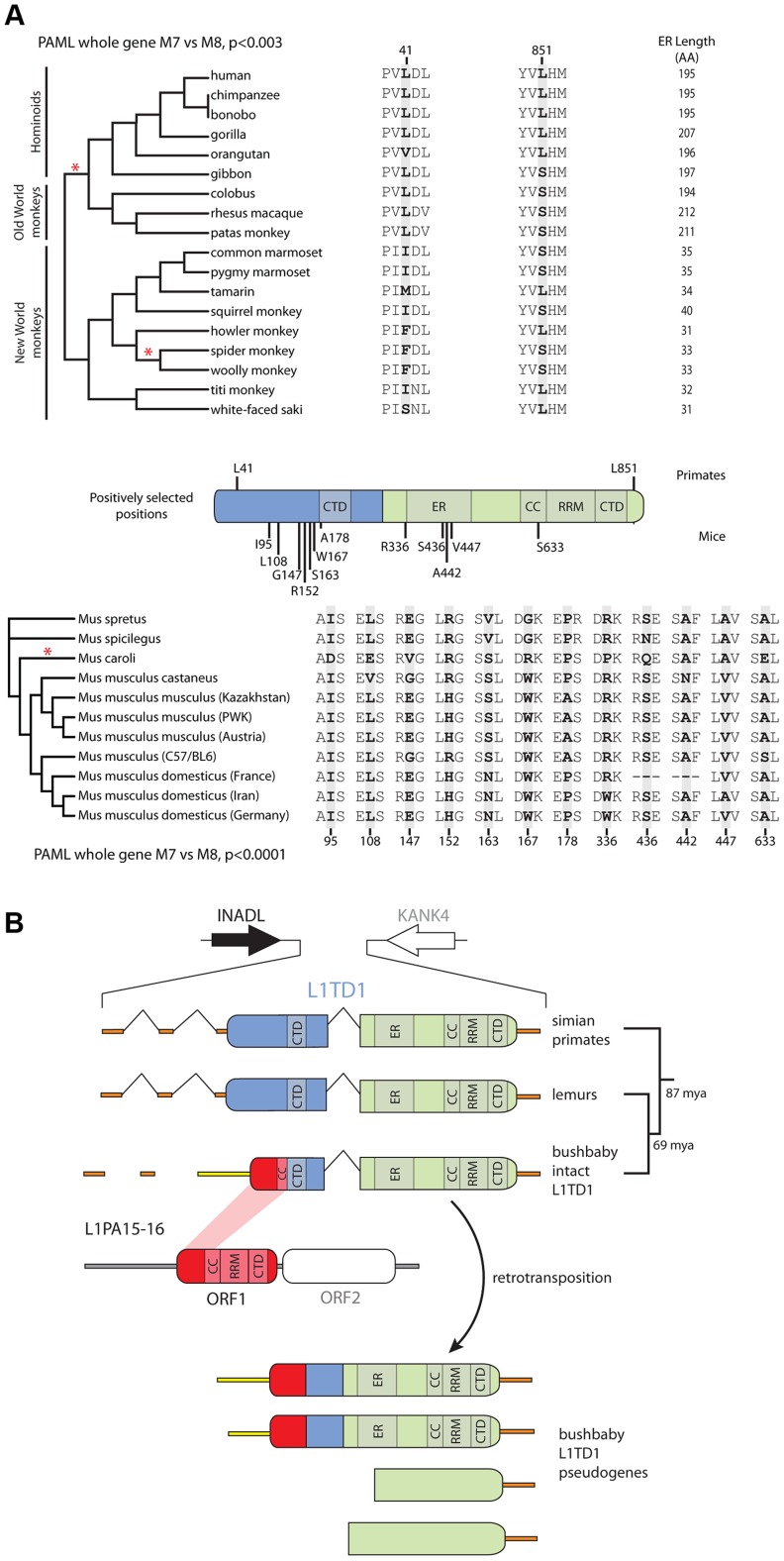
Novelty in *L1TD1* of primates and mice. A. Site-specific PAML analyses reveal a signature of positive selection in *L1TD1*. The labels on the annotated schematic indicate positions that are highly likely to be evolving under positive selection (P>90%) according to PAML NSsites (Table 1) in primates and mice (above and below the gene diagram, respectively). A species tree of primates or an *L1TD1* gene tree of mice, shows branches with statistically significant episodic diversifying selection (p<0.05) according to HyPhy's Branch-site REL (marked with a red asterisk). To the right of each tree, the amino acids found at each positively selected position are shown, along with the length of the glutamic acid-rich region in each primate. Position numberings are based upon the human and *M. musculus* (C57/BL6) sequences. B. The *L1TD1* gene of bushbaby has acquired a novel 5′ end of coding exon 1 through the insertion of a portion of a L1 element from the L1PA15-16 class (shown in red). The gene retains high sequence conservation with *L1TD1* of lemurs and simian primates across the latter half of its first coding exon and all of its second coding exon (shown in blue and green). This insertion is unique amongst all the species we have examined, and is not evident in lemur genomes (mouse lemur or aye-aye). Elsewhere in the genome, bushbaby contains at least two complete and two partial processed *L1TD1* pseudogenes that allowed us to infer the structure of the active *L1TD1* gene.

Due to the finding of positive selection on primate L1 ORF1p [38] and the availability of multiple primate genome assemblies and DNA samples, we first focused on characterizing signatures of evolutionary selection in *L1TD1* of primates. Through database searches and PCR-based sequencing of genomic DNA samples, we assembled 18 *L1TD1* sequences spanning the simian primate phylogeny, representing more than 40 million years of evolution (Figure 4A). Consistent with *L1TD1*'s important role in pluripotency maintenance in human ES cells, we found no instances of *L1TD1* loss or pseudogenization in primates. Overall, we found a high degree of conservation in *L1TD1* in primates, with average dN/dS of ∼0.5 over the primate phylogeny. dN/dS is a normalized ratio indicating whether amino acid-altering evolutionary changes occur more often than expected given the rate of neutral mutations, with values <1 indicating overall conservation, and values >1 indicating overall positive selection.

Though we observe an overall signature of purifying selection in *L1TD1*, there could nonetheless be signatures of diversifying selection occurring on just a few domains or residues. To assess whether positive selection has acted on primate *L1TD1*, we used maximum likelihood methods (NSsites models in the PAML suite [39]) to test for positive selection at individual codons. We found that a model permitting positive selection in the alignment fit the primate data significantly better than models that disallow positive selection (M8 vs. M7, M8 vs. M8a, p<0.01). Both coding exons show evidence of positive selection, suggesting that both domesticated ORF1p-like regions have been recurrently selected for functional novelty. Further, the positive selection is remarkably localized. Only a few *L1TD1* codons (∼1%) show a signature of positive selection, but these codons have a high average dN/dS (>8, Table 1). Such highly localized signatures of recurrent positive selection often represent direct contacts with antagonistic entities (discussed below). We note there is no overlap between the positively selected sites in primate *L1TD1* and the regions previously found in L1 ORF1p, which are largely in the coiled-coil domain [38], [40]. FUBAR, carried out in the HyPhy suite of programs confirmed our finding of highly localized positive selection (Materials and Methods, Table 1) (Murrell, 2013 #).

**Table 1:** Primate and mouse *L1TD1* are evolving under positive selection.

					Sites identified^1^			
Species Group	Model	2Δlnλ	df	p-value^2^	Position	AA	PAML	FUBAR
Primates	M7 vs M8	11.93	2	0.0026	43	F	**0.95**	**0.98**
					156	Y	0.88	**0.95**
					173	K	0.56	**0.95**
					229	K	0.68	**0.94**
					329	G	0.71	**0.93**
					887	S	**0.91**	0.42
Mice	M7 vs M8	17.23	2	3.27×10^−8^	95	I	**0.92**	**0.91**
					108	L	**1.00**	**0.98**
					147	G	**0.99**	**0.97**
					152	R	**0.95**	0.89
					163	S	**0.99**	**0.97**
					167	W	**0.93**	0.88
					178	A	**0.95**	0.89
					336	R	**0.94**	**0.91**
					346	E	0.85	**0.91**
					436	S	**1.00**	0.62
					442	A	**0.93**	**0.91**
					447	V	**0.90**	**0.90**
					530	G	0.89	**0.91**
					633	S	**0.91**	**0.90**

1 Codon positions are derived from the full-length translation-based alignments, and amino acids are those found in the spider monkey or NCBI mouse reference sequence. Sites with a high posterior probability of positive selection in PAML (Bayes Empirical Bayes, P>0.9) or FUBAR (Empirical Bayes, P>0.9) analyses are shown in bold.

2 The PAML p-value was calculated using twice the difference in log-likelihood between models M7 and M8 and two degrees of freedom. PAML analysis was carried out using the F61 model of codon frequencies, but similar results were obtained for the F3×4 model and various initial omega values.

Since we hypothesize that the ancestral function of *L1TD1* may have been genome defense, we expect that *L1TD1* should have evolved under positive selection within many branches of the mammalian phylogeny. To generalize our finding of pervasive positive selection in the primates, we analyzed the evolution of *L1TD1* within the genus *Mus*. We assembled 10 complete *L1TD1* sequences from databases, PCR-based sequencing, and RNA-seq data. Again, all species contained an intact coding sequence with a high degree of conservation (average dN/dS  = 0.46). However, similar to the primate analysis, PAML NSsites found a small proportion of sites with a high average dN/dS (∼3%, dN/dS >12), as well as a highly significant gene-wide signature of positive selection (Figure 4A; M8 vs. M7, M8 vs. M8a, p<0.01). We also found statistical support using FUBAR for 8/12 positions identified as positively selected by PAML (Table 1; PAML M7 vs M8 BEB, P>0.9; FUBAR P>0.9). To eliminate any false positive selection signals that could arise from phylogenetic discordance, we used the HyPhy program GARD to identify potential recombination breakpoints in the *Mus L1TD1* alignment [41]. The alternative phylogenies given by GARD attempt to correct for any recombination that may have occurred and given rise to a scenario where no single tree accurately fits the entire *L1TD1* sequence. While GARD found no statistically significant breakpoints according to the KH test, we nonetheless tested whether *Mus L1TD1* retained a statistically significant signature of positive selection integrating the generated alternative phylogenies (Figure S1). Using the GARD-generated trees, we still observed a strong signature of gene-wide positive selection according to PAML (p<0.01) and FUBAR still identified 7 positively selected positions.

In addition to phylogeny-wide selection at a few specific codons, branch-specific analyses of dN/dS ratios highlighted episodic positive selection along a few specific branches of the primate phylogeny including the branch leading to the common ancestor of spider monkey and titi monkey, as well as the branch leading to the OWMs and hominoids (Figure 4A, red asterisks, Branch-site REL, p<0.05) [41]. Using PAML, the branch leading to the common ancestor of gorilla, human and chimpanzees (after the split from orangutan) showed a whole gene dN/dS of 2.87 (1 synonymous change and 11 nonsynonymous changes); the branch preceding it shows a whole gene dN/dS of 1.671 (1 synonymous change, 5 nonsynonymous changes). While neither of these ratios is significantly greater than one, it is notable that these two branches span a time window ∼9–20 Mya, overlapping the ∼40–12 Mya time period in the lineage leading to humans shown to exhibit positive selection in L1-ORF1 and of particularly intense L1 activity [38].

Our finding that *L1TD1* has evolved under positive selection in primates and mice would be unexpected if its sole function was in pluripotency; we would expect such genes to be highly conserved. We excluded an intriguing alternative possibility that genes involved in pluripotency might not be evolving as slowly as one would intuitively assume. In a genome-wide analysis of dN/dS values calculated from trios of human-chimpanzee-macaque orthologs [42], genes identified in a screen for determinants of ES cell identity [43] are indeed evolving more slowly than control genes (Wilcoxon p = 0.008; Figure S2). Thus, our finding of positive selection in *L1TD1* is indeed unexpected if its only role were in pluripotency maintenance.

We cannot evaluate the extent to which positive selection has shaped protein regions that have experienced length-changing insertion-deletion changes, because they are unsuitable for codon-based analyses of positive selection. However, we note significant divergence in the glutamic-acid rich region of *L1TD1* that separates the two L1 ORF1p homology regions. For instance, New World monkeys have a deletion of ∼140 amino acids at the beginning of *L1TD1* coding exon 2 relative to the Old World monkeys and hominoids. The functional significance as well as the selective pressures that might have driven these changes is unknown; it is quite possible that these changes are completely neutral and have little impact on *L1TD1* function.

We also discovered a substantial restructuring of the *L1TD1* gene of bushbaby (*Otolemur garnettii*) that may have a significant impact on its function. In the bushbaby genome, we found that the latter two-thirds of the *L1TD1* coding region are completely typical and align well with orthologous sequences from other primates and mammals, with no stop codons or frameshifts. However, the first one-third of bushbaby *L1TD1* is not orthologous to any other *L1TD1* (Figure 4B). Instead, it appears the bushbaby *L1TD1* has acquired an entirely novel 5' end. Two processed *L1TD1* pseudogenes in the bushbaby genome also include this novel 5' end. Since processed pseudogenes lack introns, they provide independent confirmation of the transcript structure of *L1TD1*. On closer examination, the novel 1400 nucleotides at the beginning of the bushbaby *L1TD1* includes ∼360 nucleotides of protein-coding sequence from ORF1p of a L1 element of the L1PA15-16 class, a primate-specific L1 element. The remaining ∼1000-nucleotide 5′ UTR region comprises a patchwork of several other repetitive elements, and likely arose by a series of nested insertion events. Although the repetitive element portions of this 5' UTR are found in several other loci in the bushbaby genome, the only places they are found in this particular combination are at the *L1TD1* syntenic locus and in the *L1TD1* processed pseudogenes (Figure 4B). Thus, it appears that bushbaby *L1TD1* has undergone a recent remodeling, replenishing its N-terminal region with a more current version of a L1 element than the version captured in the original domestication event ∼100 million years ago. The newly replenished version of bushbaby *L1TD1* has evolved under purifying selection (Figure S3), ruling out the alternate possibility that this *L1TD1* rearrangement is a pseudogenization event. This novel *L1TD1* structure is not evident in the genomes of two lemur species, the gray mouse lemur (*Microcebus murinus*) and the aye-aye (*Daubentonia madagascariensis*), which both encode a more typical *L1TD1*, and we have not observed evidence of bushbaby-like *L1TD1* restructuring in the any other mammalian genomes examined (Figure 3). This implies that the bushbaby *L1TD1* remodeling occurred after the split between the lemur and bushbaby lineages, nearly 60 million years ago. Closer examination of other prosimian lineages will allow a more precise dating of this event.

From these forms of variation in the primate and mouse lineages, we conclude that genetic innovation has been adaptively selected for in *L1TD1* through a number of mechanisms including mutation of individual amino acids and perhaps expansions and contractions of the glutamic acid-rich region. Importantly, the fixation of a new gene structure in the bushbaby derived from a recently active L1 suggests selection for functional novelty through yet another L1-derived sequence. The signature of positive selection in both mice and primates suggests *L1TD1* may be coevolving with some pathogen. Intriguingly, a strong signature of positive selection, indicative of such ‘arms-races’, has been previously seen in primate L1 evolution, with an especially striking signature in ORF1p [38], [40]. Combined with its origin as a domesticated L1 gene and the novelty in bushbaby (which further emphasizes the selection pressure to maintain the L1-like character of *L1TD1*), we considered whether *L1TD1* could be engaged in a genetic conflict with L1 or some other retroelement. This function could parallel the known role of another domesticated retroelement gene, *Fv1*, in defense against elements similar to its progenitor [13], [14]. To more clearly understand this hypothetical relationship, we reasoned that genes that function solely in L1 restriction might be lost in species where active L1s have previously been shown to have gone extinct – the megabats and the rice rats.

### *L1TD1* and the loss of L1 activity in megabat genomes

**Figure 5:**
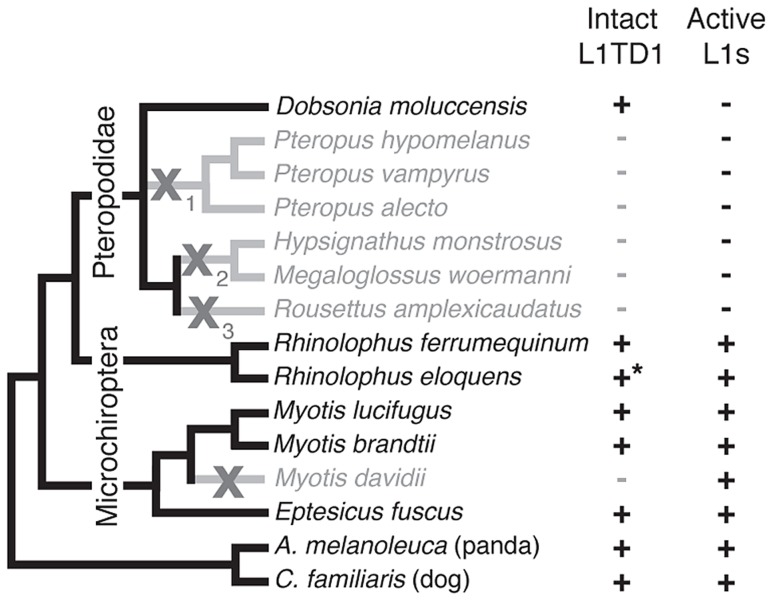
Loss of *L1TD1* in megabats appears to follow the loss of L1 activity. We obtained *L1TD1* sequences from thirteen bat species (Materials and Methods). We show a species tree partly based upon a published megabat phylogeny [82], inferring placement of additional taxa using species from the same genus. In addition, we used our *L1TD1* sequences to resolve relationships in the *Pteropus* and *Myotis/Eptesicus* clades. Species in which *L1TD1* appears intact are shown in black, and those in which *L1TD1* harbors inactivating mutations (stop codons, frameshifting insertions/deletions) are shown in gray. Some species share the same inactivating mutation(s) represented by the subscripts of the gray X symbols, suggesting *L1TD1* was lost three independent times in the megabats. For one species, *R. eloquens* (starred), we were only able to obtain part of coding exon 1 of *L1TD1*, but this region is intact. Presence or absence of active L1s is based upon previous data [44] and analysis of the *M. davidii* genome assembly (Yang and Wichman, unpublished data).

Previous studies have demonstrated that a number of megabat species lack active L1 elements. Indeed, it is likely that L1s experienced an extinction event in the megabat ancestor between 24 and 58 million years ago [44]. We decided to explore the potentially antagonistic relationship between *L1TD1* and L1 by looking in bat species where the most detailed studies of L1 extinction have been completed. In addition to identifying *L1TD1* sequences from seven available chiropteran genome sequences, we amplified and sequenced the *L1TD1* coding exons from six additional bat species (Figure 5). We were able to unambiguously determine the complete coding sequence of *L1TD1* for twelve bat species; in the thirteenth species, *Rhinolophus eloquens*, we were only able to obtain part of coding exon 1 of *L1TD1*.

Bats (Chiroptera) are divided into the monophyletic megabat clade Megachiroptera and a polyphyletic microbat clade, the Microchiroptera. Among the Microchiroptera, our survey revealed yet another case of an independent *L1TD1* loss, in the genome of the microbat *Myotis davidii* (Figure 5). In this genome, our bioinformatics analysis (Yang and Wichman, unpublished data) shows that the most recently inserted L1s have ∼99.5% identity to each other and retain their open reading frame; further, these highly similar sequences maintain conservation of amino acids known to be completely conserved across young mammalian L1s [45], suggesting L1s are still active in the *M. davidii* lineage. This situation parallels that of Cetartiodactyla and Afrotheria, which have both lost *L1TD1* but likely maintain active L1s.

In contrast, among the megabats, we found that most species that have lost active L1s have also lost *L1TD1*. The only exception was the megabat species *Dobsonia moluccensis*, which has lost active L1s but possesses an apparently intact *L1TD1* that has evolved under purifying selection for at least some of the time since it diverged from other megabats (Figure S4). Our observation of unique inactivating coding mutations among subsets of the megabat species suggests there were at least three independent losses of *L1TD1* (Figure 5, X's with subscripts). However, due to the poor resolution of the phylogenetic relationships of the other six megabat species with *Dobsonia moluccensis* (Figure 5), we cannot formally rule out the alternate possibility that the initial loss of *L1TD1* function occurred once, via a non-coding mutation. Under both scenarios, we infer that the extinction of L1s preceded loss of *L1TD1* in the megabats [44].

Thus, it appears that loss of L1 may have led to loss of *L1TD1* in megabats except in *Dobsonia moluccensis* (see Discussion). This evolutionary relationship between L1 elements and *L1TD1* is consistent with the possibility that *L1TD1* was domesticated by mammalian genomes to antagonize L1s, similar to the domestication of *Fv1* for defense against retroviruses. Under this model, loss of active L1s would relieve the selective pressure to maintain *L1TD1* in megabat genomes.

### *L1TD1* and the loss of L1 activity in rice rat genomes

In addition to the loss of active L1s in the megabats, a group of rice rats in the subfamily Sigmodontinae are the only other group of mammals in which a loss of active L1s has been well characterized [45]. To investigate whether these species might exhibit a correlation similar to that of the megabats, in which L1 loss and *L1TD1* loss co-occur, we sequenced the *L1TD1* gene from ten L1-less Sigmodontinae species (5 complete sequences, 4 coding exon 1s, 1 coding exon 2), as well as two Sigmodontinae species that retain active L1s (*Oecomys bicolor* and *Reithrodontomys fulvescens*). In all cases, we found an intact coding sequence, suggesting these species have retained a functional copy of the *L1TD1* gene (Dataset S1).

We suggest two hypotheses for the apparent retention of *L1TD1* in these species, despite the absence of active L1s. First, it is possible that not enough time has passed for inactivating mutations to appear in the coding sequence of *L1TD1* in these species; we note that the loss of active L1s in these species is much more recent than the loss of L1s in megabats (7.2–12.3 million years ago in the rodents vs 24–58 million years ago in the megabats). While we cannot definitively rule out the possibility that *L1TD1* is neutrally evolving within these species, overall the tree is better fit by a single purifying dN/dS than by a dN/dS fixed at 1 (p<0.001). Further, most of the Sigmodontinae sequences exhibit pairwise dN/dS ratios that suggest purifying selection within the clade (Table S1). We therefore favor the second possibility that retention of a functional *L1TD1* in these species results from selection pressures independent of L1s. For example, *L1TD1* could play an essential role in pluripotency (despite the dispensable role of the *Mus L1TD1*). Intriguingly, despite having an overall tree length similar to our *Mus* alignment, we do not find any evidence of positive selection in the *L1TD1* genes from Sigmodontinae using PAML NSsites on either full sequences (M7 vs M8, p = 0.33), coding exon 1 alone (p = 0.53), or coding exon 2 alone (p = 0.84). Alternatively, *L1TD1* in sigmodonts could play a role in genome defense against another element active in these genomes. Indeed, the sigmodontine rodents have a highly active family of ERVs, the mysTR elements [46]. Expansion of this family preceded the death of L1s, but these elements are very active, with 3500 to 7000 species-specific insertions in the L1-extinct species examined [47]. This recent ERV amplification in Sigmodontinae contrasts with the megabats (where *L1TD1* has been lost in many species); there are apparently no highly active DNA or RNA elements in megabats [48]. If *L1TD1* can suppress retroelements other than L1s, this could explain why the gene is retained in sigmodontine rodents but not in megabats.

While these results do not provide additional support for our proposed correlation between L1 loss and *L1TD1* loss, they do provide another example of the potentially stochastic co-option of *L1TD1* into essential cellular processes that has driven the variable gain and loss of this gene throughout mammalian evolution.

## Discussion

Domesticated genes such as *L1TD1* provide a window into the opportunistic means by which host genomes can exapt new genetic functions from their resident mobile elements. L1 elements and non-autonomous SINE elements that rely on L1 for mobility comprise a substantial fraction of the human genome (at least 17 and 30 percent, respectively [49]). This abundance, together with a relatively long history of vertical transmission has provided ample opportunities for host genome domestication of L1 elements. Indeed, we see evidence of this exaptation for enhancer functions, in exonization of Alu elements and small L1 fragments, and even in long non-coding RNAs [4], [6], [21]–[23], [25]–[28]. However, *L1TD1* is unique in being the only known host protein-coding gene whose protein coding sequence is derived almost entirely from the coding sequence of an L1. In addition, its exclusive expression in stem cells and the germline and the observation that *L1TD1* appears to be required for pluripotency maintenance in human ES cells [31], make it a powerful example of retroelement domestication for an essential function. Exaptation for an important function such as pluripotency maintenance draws immediate parallels to other retroelement domestications such as the retroviral envelope-derived *syncytin* genes and the sushi-retroelement derived *Peg10*-related genes, both of which serve essential roles in mammalian placentation [7], [11], [12]. Indeed, at first glance, our estimate of the evolutionary age of *L1TD1* (only slightly younger than *Peg10* and older than any identified *syncytin* gene) does suggest long-term preservation for some important function.

Nonetheless, our elucidation of *L1TD1*'s evolutionary history challenges these parallels. For instance, we found multiple instances of *L1TD1* loss or pseudogenization at different stages of mammalian evolution, in contrast to *Peg10*, which is intact in all examined mammals [11], [50], [51]. Furthermore, *Peg10* is subject to strong purifying selection (unpublished data), presumably due to the constraint imposed by its essential functions in placentation and genomic imprinting. In contrast, we have found evidence of diversifying selection acting on *L1TD1* in primates and mice, along with genetic innovation in bushbaby via a partial replacement of *L1TD1* coding sequence with the ORF1 of a younger L1 element. This pattern of genetic innovation and sporadic loss is more reminiscent of another class of domesticated retroelement genes, exemplified by the *Fv1* retroviral *gag*-derived gene in *Mus* species, which serves as a form of host defense against a broad array of incoming retroviral capsids [15], [18].

It is noteworthy that germline and early embryonic tissues represent the primary battleground for the conflict between hosts and L1 elements. In order to propagate new copies to future generations, L1s must successfully retrotranspose in these cells. However, these are the very compartments where a host could incur the greatest fitness cost from L1 replication. The highly specific expression pattern of *L1TD1* suggests that its target could be genomic retroelements that mobilize during germline development and early embryonic stages.

Three findings lead us to consider the intriguing speculation that L1TD1 may target L1 elements themselves (although other functions for *L1TD1* are quite possible). First, we find that extinction of L1 activity appears to have been followed by *L1TD1* loss in several megabat lineages. This relationship between L1 loss and *L1TD1* loss is far from perfect. For example, in the microbat *Myotis davidii* and other mammalian genomes (Afrotheria and Cetartiodactyla), *L1TD1* loss is not accompanied by L1 extinction. This is easily explained, because a number of different restriction factors exist to defend against L1 in mammalian genomes [52], and in particular genomes, the previously beneficial function of *L1TD1* may become redundant and dispensable. Also, if a rapidly evolving L1 were to escape restriction by *L1TD1*, control over this rogue element could be regained through adaptive mutations in restriction factors besides *L1TD1*, thereby relaxing selective pressure to maintain *L1TD1*. Alternatively, L1 retrotransposition rates are known to have varied over evolutionary time: periods where L1 retroposition rates are low could also lead to relaxed selective pressures on *L1TD1* retention. A similar relaxation of selective pressure has been invoked to explain the idiosyncratic loss of *Fv1* in *Mus* species [15]. Second, we found that bushbaby *L1TD1* underwent a partial replacement with a more current version of L1 ORF1. Although such ‘replenishment’ could represent a neutral event unique to the bushbaby, the fact that it has occurred suggests a model in which the ‘newer’ version of *L1TD1* was fixed in the bushbaby genome because it conferred a selective advantage over the pre-existing version, which itself formed from a much more ancient L1 domestication. Third, our observation that *L1TD1* has experienced positive selection in primates (and mice) is consistent with previous findings that L1 ORF1 has evolved under positive selection during primate evolution [38], [40]. Like *Fv1* action against distantly homologous retroviral capsids, the observed positive selection suggests the possibility that *L1TD1* may be in direct conflict with L1 ORF1p from active L1 elements. Since L1 elements also provide the machinery for non-autonomous elements like SINEs [53] and likely HAL1s [54], it is also possible that these elements represent the true targets of *L1TD1*, rather than L1 itself.

L1TD1 could function as a L1 restriction factor through a number of possible mechanisms. First, analogously to *Fv1*, L1TD1 might interfere with the homotrimerization of L1-ORF1p that is necessary for L1 retrotransposition. This trimerization normally happens through ORF1p's coiled-coil domain [55]. We note that *L1TD1* coding exon 2 has preserved its coiled-coil domain, which may be necessary for this interaction. Under this model, L1TD1 could act as a dominant negative version of ORF1p. Its binding to ORF1p could lead to L1 restriction, which could then drive episodes of diversifying selection in L1 ORF1p to escape L1TD1 restriction [40]. In response to these escape mutations in L1, we might expect *L1TD1* to rapidly evolve to restrict the variant L1 – a situation consistent with the positive selection we observe in *L1TD1*. Another possibility is that L1TD1 could outcompete L1 ORF1p for binding to the ORF2p protein, the likely rate-limiting component for retrotransposition. Alternatively, L1TD1's RNA-binding activity could be the source of its restrictive abilities. In this model, L1TD1 could act as a competitive inhibitor of L1 ORF1p, binding the RNA of transcribed retroelements and blocking the downstream pathway that would normally create new copies. This could function through simple titration or through a more complex mechanism whereby L1TD1 localizes L1 RNA to P-bodies [32] for sequestration or decapping and subsequent degradation. The previously reported association of L1TD1 with the TRIM28 (KAP1) complex [56] suggests yet another possible mechanism of restriction. TRIM28 is known to function in the silencing of exogenous and endogenous retroviral integrations by recruitment of heterochromatin proteins to some retroviral elements [57]. Under this epigenetic silencing model, L1TD1 may function in an analogous role to the zinc finger adaptor proteins that help target TRIM28 and consequently the silencing complex to particular genomic sequences. Thus, constant adaptation to bind either DNA or RNA at L1 integration sites could drive diversifying selection of *L1TD1*. It is intriguing to imagine a model wherein the silencing machinery could target any genomic parasite through a modular adapter protein that recognizes the newly integrated sequence (although it must be noted that such activities have not been ascribed to L1 ORF1p from which *L1TD1* derived). Further characterization will shed light into whether L1TD1 encodes any or all of these biochemical activities.

When we examined the genome sequences of bats, we found that many species that have lost active L1s have also lost *L1TD1*. However, *Dobsonia moluccensis* still maintains *L1TD1* as an intact protein-coding gene, despite the fact that this species lost active L1s many million years previously. Similarly, we found *L1TD1* intact in the subset of the Sigmodontinae rodents previously shown to have lost active L1s [45]. This implies that the proposed selection for restricting active L1 elements cannot be the only constraint that dictates retention of *L1TD1*. We hypothesize that idiosyncratic, highly divergent L1 insertion patterns in different mammalian genomes could provide an explanation of both the retention of *L1TD1* in mammals without an active L1 and the essential role in pluripotency maintenance that *L1TD1* plays in humans. Under this model, *L1TD1*'s role as either important or dispensable for pluripotency maintenance depends on the pattern of where L1s have inserted in the genome of each species. In various contexts, transposable elements have been shown to affect the expression of genes in their vicinity [58], [59], and some elements are included in the untranslated regions of host mRNAs [60], [61]. If L1TD1 transcriptionally or post-transcriptionally silences L1 elements, this silencing could alter the expression of host genes near L1s, or with L1s in their UTRs. Under this model, in some mammalian genomes, L1 insertions would not be in proximity to genes that impact the pluripotency program (Figure S5). In these species, extinction of L1 would result in the loss of constraint on *L1TD1* (e.g., megabat *P. vampyrus*), and experimental knock-down of *L1TD1* would have no effect on pluripotency maintenance (e.g., *M. musculus* [33]). In contrast, in other mammalian genomes where L1 insertions occurred near genes whose repression is important for pluripotency maintenance, loss of *L1TD1* would lead to loss of pluripotency (e.g., *H. sapiens* [31]). In fact, repression could have been incorporated into any number of functionally important pathways in the cells where *L1TD1* is expressed, simply depending on which genes L1s landed next to during evolution. We hypothesize that in such species, extinction of L1 activity would not relax the selective pressures to maintain *L1TD1* (e.g., megabat *D. moluccensis*, Sigmodontine rice rats), which would now be required to recognize ‘dead’ L1 copies in order to maintain these repressive programs.

Although we present the hypothesis that *L1TD1* was originally domesticated as an anti-L1 restriction factor and was subsequently recruited for pluripotency regulation in humans by virtue of direct repression of L1 elements near functionally relevant genes, this idea is at present highly speculative. L1TD1 has experienced a long evolutionary history functioning in the context of other factors expressed in pluripotent cells, and it is possible that L1TD1 could have been coopted by these factors. For instance, L1TD1 could be involved in regulating RNA involved in pluripotency via its ancestral chaperone function, or in regulating important protein complexes via protein-protein interactions. We look forward to experimental investigation of all of these hypotheses; none of them can completely explain all of our evolutionary observations (*L1TD1* loss and retention in various lineages, positive selection and remodeling in bushbaby), perhaps suggesting that L1TD1's function has changed multiple times over the course of mammalian evolution.

In summary, we posit that *L1TD1*'s original function could have been in genome defense (similar to *Fv1*), and that it still has defense functions in many mammalian species where it is retained (including humans). We note that our evidence for *L1TD1* diversifying selection (and inferred genome defense) comes in part from the primate lineage, which is also where its role in pluripotency maintenance is best established. Later in evolution, by virtue of either its transcriptional or post-transcriptional silencing of L1, L1TD1 may have become intricately enmeshed into the transcriptional program of L1-proximal genes in some species, meaning that *L1TD1* also acquired an absolutely essential function (similar to *Peg10* and *syncytin*). An interesting feature of this model is that L1TD1's essential function (or lack thereof) is thus a consequence of stochastic L1 insertion patterns in different mammalian genomes. This stochasticity could provide an explanation for why *L1TD1* has been lost in some species, and why it is responsible for pluripotency maintenance in only some mammalian genomes.

## Materials and Methods

### Ethics statement

The animals used in this study are wild-derived laboratory animals of the species *Mus musculus*, *Mus spretus*, *Mus spicilegus*, *Mus mattheyi* and *Apodemus uralensis*. None of these species are protected. Permits for catching the founding members of each line were not required at the time they were caught. Some specimens were caught on the properties of private landowners, with their oral permission to enter the property and catch mice. All animal work was carried out by experienced personnel at the Max Planck Institute for Evolutionary Biology, following the legal requirements in accordance with German animal welfare law (Tierschutzgesetz) and FELASA guidelines. Permits for keeping mice were obtained from the local veterinary office “Veterinäramt Kreis Plön” (permit number: 1401-144/PLÖ-004697). The dissection of animals and organ extractions were performed according to the German Animal Welfare Act § 8a Abs. 1 Nr. 3b TierSchG; ‘Organ-/Gewebsentnahme zu wissenschaftlichen/diagnostischen Zwecken TierSchG' V 312-72241.123-34.

### Biological materials

Sigmodontine rodent liver tissue was obtained on loan from the Museum at Texas Tech University (Lubbock, TX).

For *Mus* samples, mice of different ages were sacrificed by CO_2_ asphyxiation followed by cervical dislocation. Mice were then dissected and tissues were snap-frozen within 5 minutes post-mortem. Liver (front view: front left lobe), both testes, and whole brain including brain stem were collected. For the outbred populations, Iran (AH), France (M), and Germany (CB) for *Mus musculus domesticus*, and Austria (WI) and Kazakhstan (KH) for *Mus musculus musculus*, eight individuals each were sampled. For inbred groups, *Mus musculus castaneus* (TA), *Mus spretus* (SP), *Mus spicilegus* (SC), *Mus mattheyi* (MA) and *Apodemus uralensis* (AP), four individuals each were sampled. All mice were obtained from the mouse collection at the Max Planck Institute for Evolutionary Biology.

### *Mus* transcriptome sequencing, processing, and mapping/assembly

The sampled tissues of each *Mus* group were used for RNA extraction with the RNAeasy Kit (Qiagen) and pooled at equimolar concentrations. Quality of the RNA was measured with BioAnalyzer chips (Agilent), for the individual samples and pools, and samples with RIN values below 7.5 were discarded. The pools were subsequently submitted to the Cologne Center for Genomics (CCG) for further processing and sequencing. The sequencing of the samples was performed using a polyA tail purification step, followed by cDNA synthesis, Illumina library preparation, and sequencing with an Illumina HiSeq 2000 sequencer. Each transcriptome sample was sequenced in approximately one third of a HiSeq2000 flow-cell lane (one flow-cell lane per taxon).

All raw data files were trimmed for adaptors and quality using Trimmomatic [62]. The quality trimming was performed base-wise, removing bases below quality score of 20 (Q20), and keeping reads whose average quality was of at least Q30. Reads whose trimmed length was shorter than 40 bases were excluded from further analyses, and pairs missing one member because of poor quality were also removed from any further analyses.

Quality-filtered transcriptome reads were aligned against the mm10 version of the mouse reference genome from UCSC [63] using NextGenMap [64]. Reads which were ambiguously or poorly mapped (MAPQ <20) were removed from the analyses. Quality-filtered transcriptome reads for each taxon were merged into a single input file, discarding tissue information, and assembled de novo with the Trinity platform [65] using default parameters.

### Sequence collection

*L1TD1* sequences were obtained from publicly available primate genome databases using PSI-BLAST [66] against the NR database or TBLASTN [66] against the HTGS database, with human *L1TD1* as a search seed (Table S2). For sequencing of other primate, Sigmodontinae, and *Mus* species (Table S2), exon 1 and exon 2 were PCR amplified individually from genomic DNA using oligonucleotides designed against intronic regions. PCR primers were designed against intronic regions neighboring each exon based upon an alignment of mouse, rat, and Chinese hamster for rodents or human, rhesus, and squirrel monkey for primates (Primates Exon1 Sense: CAGAATCCAGTCTTGACAACATATCC; Primates Exon1 Antisense: CAGGAGAATCACTTGAACCTGGG; Primates Exon2 Sense: GTCAGAATGGAAGCCATATTAAAATTAGTG; Primates Exon2 Antisense: GCTATTAGCTGTCCATCCTTCTGG; Rodent Exon1 Sense: GYAAGWAMAYTTTCATTTGYTTATAKTTC; Rodent Exon1 Antisense: CCYATCARTYTCTRGAACYCCYRTCAARC; Rodent Exon2 Sense: GGMAAGYATACTAAATTYAGAGGGTRAAATAG; Rodent Exon2 Antisense: AASTCAACCAACMYKCAGRKAGTK). PCR products were sequenced using standard Sanger sequencing.

For *Mus* sequences, informative reads were obtained with samtools [67] from alignments overlapping with the *L1TD1* annotation. Known sequences from the coding sequences of *L1TD1* were used to identify the most similar assembled contigs in each taxon using nucleotide-nucleotide blast [68] (e-value <1e-10).

*L1TD1* sequences from primate, Sigmodontinae, and *Mus* species have been deposited in Genbank under accession numbers KJ994281-KJ994329.

### Alignments and positive selection analysis

Primate and *Mus* nucleotide sequences were aligned using the ClustalW ‘translation align’ function in Geneious Pro (Biomatters Ltd.). Alignments were refined manually, including truncation of the poorly aligned glutamic-acid rich region, and this alignment and an established primate phylogeny [69] or *Mus L1TD1* gene tree were input into the CODEML sites model of PAML [70] to detect positive selection at individual sites. Positively selected sites were classified as those sites with a M8 Bayes empirical Bayes posterior probability >90%. FUBAR was performed using the web-based implementation of HyPhy (www.datamonkey.org) [71], [72]. To test for signatures of positive selection along individual branches of the primate phylogeny, we used Branch-site REL in HyPhy [41] or the branch model of PAML. For PAML, the statistical significance of any branch that showed dN/dS >1 in the free ratio model (model  = 1) was tested using a two-ratio tree (model  = 2) by specifying the branch of interest as a foreground branch and all other branches as background branches. We then compared the likelihood of a model where the foreground branch had a freely estimated dN/dS with the likelihood of a model where that branch had dN/dS fixed at the neutral value of 1 [39].

For the analysis shown in Figure S2, we obtained dN/dS values from a genome-wide analysis of trios of human-chimpanzee-macaque orthologs [42], made available via Adam Siepel's website (http://compgen.bscb.cornell.edu/orthologs). To be conservative, we filtered out dN/dS values that might be artificially high for technical reasons: we eliminated values calculated from alignments of fewer than 100 codons, eliminated alignments that contained fewer than 5 evolutionary changes of any type, and included only RefSeq genes and not other genes derived from less confident annotation sets. In this dataset, alignments with no synonymous changes had apparent dN/dS values of 999; again, to be conservative, we replaced dN/dS values for these genes with a more conservative estimate of dN/dS = 2. We converted sequence identifiers to gene symbols using Bioconductor [73], allowing us to cross-reference dN/dS values with genes identified in a screen for ES cell determinants [43]. For the plot shown in Figure S2, we selected a list of 127 ES cell determinants validated by secondary screening using three markers of stem cell identity [43]; other overlapping lists of validated hits from this screen show similar evolutionary patterns.

### Mammalian sequence collection and alignment

To identify previously unannotated copies of *L1TD1* in sequenced genomes, the genomic sequence between flanking genes *INADL* and *KANK4* was extracted and compared with the corresponding human genomic region (*INADL*-*L1TD1*-*KANK4*) using Dotter [74]. Visual inspection of Dotter output enabled extraction of L1TD1 protein-coding sequences. Multispecies *L1TD1* alignments were generated using CLUSTALW [75] with manual adjustment. For all mammals mentioned in the text, genome sequences were searched for non-syntenic and/or pseudogene copies of *L1TD1* using TBLASTN with a RepeatMasked version [48] of human and mouse *L1TD1* sequences, and in some cases *L1TD1* sequence from a more closely related species.

To identify representative L1 ORF1p sequences (Dataset S2) used to build the tree shown in Figure 2, we first selected a small number of L1 ORF1p consensus sequences from RepBase [76] and obtained the sequence of an active human L1 from Genbank (LRE2, accession AAB60344.1). We then used TBLASTN [66] against whole genome assemblies (Table S2) to identify a single intact L1-ORF1p sequence from the genome assemblies of each species shown in Figure 2, selecting a copy arbitrarily from among the blast hits that did not show inactivating mutations in ORF1p.

### Phylogenetic inference

To construct the tree shown in Figure 1B, we used our alignment of *L1TD1* nucleotide sequences and the jModelTest 2 program [77] to determine that the best-fitting evolutionary model for this alignment is the GTR model with invariant sites and gamma distributed rates (GTR+I+G). We then generated a maximum-likelihood phylogeny using PhyML [78] and the GTR+I+G evolutionary model with four site categories for the gamma distribution. 1000 replicate trees were constructed, and the tree with the highest log-likelihood was chosen and displayed using MEGA5 [79].

To construct the tree shown in Figure 2, predicted protein sequences were aligned by hand (Dataset S2). We used ProtTest 3 [80] to determine that the best-fitting evolutionary model for this alignment is the JTT model with gamma-distributed rates (JTT+G). We then used PHYML to generate maximum-likelihood trees with the JTT+G model (four site categories for the gamma distribution). 1000 replicate trees were constructed, and the tree with the highest log-likelihood was chosen and displayed using MEGA5 [79]. Bootstrap values represent the percentage of trees in which the descendent taxa cluster together.

Dates of divergence mentioned in the text were generated using the TimeTree web service [81].

### Bat sequencing and database sequence collection

We obtained *L1TD1* sequences for *Pteropus alecto*, *Pteropus vampyrus*, *Rhinolophus ferrumequinum*, *Myotis lucifugus, Myotis davidii*, *Myotis brandtii* and *Eptesicus fuscus* from publicly available genome sequences. For other bat species, genomic DNA samples were obtained from tissues from The Museum, Texas Tech University. Sample accession numbers were previously published [44]. Degenerate PCR primers were designed against intronic regions neighboring each exon based upon an alignment of *P. vampyrus* and *M. lucifugus* sequences (Exon1 Sense: TTTCAGATGATTTTKTAAAWWGAKTTTRGGGG; Exon1 Antisense: TYYTMWYWAWTWAAMARSTGTTAASYYWTTSTTC; Exon2 Sense: TGGGGWTCCMAGCCTTYAAGAAMAAATC; Exon2 Antisense: CATCMCCAAGTATACTGTTAGCTGTCCATC). For *Rousettus amplexicaudatus* and *Rhinolophus eloquens* we designed a second set of primers based upon the new sequences we generated to amplify Exon1 (Sense: AAATATCACCCACATGGAAAGAATTAG; Antisense: TTCTCTTGAATCCCATATCTTCTTCC). PCR products were sequenced by Sanger sequencing. For *Nyctimene albiventer*, *Megaerops niphanae* and *Cynopterus sphinx*, we were unable to amplify or sequence any product from either exon with multiple PCR optimizations of annealing temperature, amount of genomic DNA template and concentration of magnesium ion. For two bat species, it appeared that more than one closely related *L1TD1* sequence was present. Upon cloning and sequencing multiple PCR products, we found that *M. woermanni* had 4 sequence variants and *R. amplexicaudatus* had 2 variants. For each species, at least one inactivating mutation was shared by all variants.

## Supporting Information

Dataset S1 In-frame nucleotide alignment of *L1TD1* sequences. The fasta-formatted alignment includes all *L1TD1* sequences described in the manuscript. The inactivating mutations in *L1TD1* that are shared by subsets of megabat species (Figure 5) are at the following nucleotide positions of the alignment: *P. hypomelanus*, *P. alecto* and *P. vampyrus* share three mutations: a stop codon at bp 34–36; a stop codon at bp 106–108; a 1 bp insertion at bp ∼774. *H. monstrosus* and *M. woermanni* share a different set of three mutations: a stop codon at bp 385–387; a 1 bp deletion at bp 720; a 1 bp deletion at bp 939. *R. amplexicaudatus* does not share any of the above mutations, but has numerous lineage-specific inactivating mutations.
(TXT)

Dataset S2 Amino acid alignment of L1-ORF1p and the two ORF1p-homologous regions of L1TD1. The fasta-formatted alignment was used to generate the phylogenetic tree shown in Figure 2.
(FA) Click here for additional data file.

Figure S1 Positive selection in *Mus L1TD1* is robust to alternative phylogenies from potential recombination breakpoints. We used HyPhy's GARD program to test for recombination breakpoints in the *Mus L1TD1* alignment that could give rise to false signatures of positive selection. We found three potential breakpoints, though none was statistically significant (KH test, p>0.1). To ensure that our detection of positive selection was robust to the use of these alternative phylogenies, we performed PAML NSsites on slices of the alignment corresponding to each of the GARD trees, and we used the built-in functionality of DataMonkey to use these GARD-generated trees to identify positively-selected positions using FUBAR. Top, the whole gene tree is shown in black. The three breakpoints are shown on the schematic of the *L1TD1* gene with vertical lines, and the slices they delimit are shown in different colors. Below each gene segment is shown the GARD-generated tree that best describes that region. Both the PAML NSsites signature of selection and FUBAR-identified selected sites are robust to the use of these alternative phylogenies.
(PDF)

Figure S2 Determinants of embryonic stem cell identity evolve more slowly than control genes. The boxplots show the distribution of dN/dS values for genes identified in a screen for determinants of human ES cell identity [43] and for all other genes in a genome-wide dataset of dN/dS values calculated from trios of orthologs from human, chimpanzee and macaque genomes [42]. Outliers with dN/dS>1 are omitted; gray dots represent other outliers - the large number of datapoints for "other genes" precludes visualization of individual datapoints. The stem cell determinants are evolving more slowly than other genes (Wilcoxon p = 0.008). *L1TD1*'s dN/dS value in this genome-wide dataset is shown using a red horizontal line; it is evolving faster than most other pluripotency genes. Although *L1TD1* did not meet the arbitrary threshold (Fav score <−2) used to identify stem cell determinants in the published RNAi screen, its score in the screen (Fav  = −0.90) is well below the genome-wide average, consistent with previous results that human *L1TD1* is a pluripotency factor [31], [32].
(PDF)

Figure S3 Intact *L1TD1* from bushbaby *Otolemur garnettii* has evolved under purifying selection. We used codeml's free-ratio model to estimate selective pressures on *L1TD1* on each branch of the two trees shown. Above each branch we show estimated dN/dS ratios, and in parentheses below each branch we show the estimated number of non-synonymous and synonymous changes, respectively. *L1TD1* pseudogenes are shown with their labels in gray. For three selected branches we performed likelihood tests of whether the estimated dN/dS ratio is a significantly better fit to the data than dN/dS = 1 for that branch, by assuming only two dN/dS ratios for the entire tree (one ratio for the branch in question, and one for all other branches), and comparing a model where dN/dS for the branch in question was fixed at 1 with a model where dN/dS was freely estimated. Results of those tests are shown with red superscripts. A. We examined evolution of the full-length ORF of the novel bushbaby *L1TD1* structure, comparing it to two processed pseudogenes in the bushbaby genome that arose after this novel *L1TD1* structure formed. The intact bushbaby *L1TD1* is more likely evolving under purifying than neutral selection (p = 0.0003; **). B. We examined the portion of bushbaby *L1TD1* that aligns to the ancestral *L1TD1* gene, including human and aye-aye *L1TD1* genes as outgroups. The intact bushbaby *L1TD1* is more likely evolving under purifying than neutral selection (p<10^−5^; **); there is also weaker support (p = 0.07; *) for purifying selection on the shared ancestor of the intact bushbaby *L1TD1* and bushbaby processed pseudogene B.
(PDF)

Figure S4 Intact *L1TD1* from megabat *Dobsonia moluccensis* has evolved under purifying selection. We used codeml's free-ratio model to estimate selective pressures on *L1TD1* on each branch of the species tree shown. Above each branch we show estimated dN/dS ratios, and in parentheses below each branch we show the estimated number of non-synonymous and synonymous changes, respectively. Species in which *L1TD1* is a pseudogene are shown with their labels in gray. For three branches, we performed likelihood tests of whether the estimated dN/dS ratio is a significantly better fit to the data than dN/dS = 1 for that branch, by assuming only two dN/dS ratios for the entire tree (one ratio for the branch in question, and one for all other branches), and comparing a model where dN/dS for the branch in question was fixed at 1 with a model where dN/dS was freely estimated. Results of those tests are shown with red superscripts: the *Dobsonia moluccensis* branch is more likely evolving under purifying than neutral selection (p = 0.003), as is the branch ancestral to *D. moluccensis* and the three *Pteropus* species (p = 0.002). In contrast, for the *Pteropus alecto* branch, a neutral model is as good a fit to the data as a model invoking purifying selection (denoted by n.s., for non-significant).
(PDF)

Figure S5 *L1TD1* could become essential based upon the pattern of L1 insertions in a specific genome. We present a scheme under which L1TD1 could idiosyncratically adopt a pluripotency role in a species-specific fashion. A. We schematize the genomes of three exemplar species with an identical stretch of seven genes (rounded boxes), one of which must be silenced for pluripotency maintenance (orange boxes). B. L1s (black rounded boxes) insert randomly into each genome. One insertion (species 3) happens to be near the silenced gene. C. According to our hypothesis, L1TD1 silences the newly inserted L1s (grayed areas), as well as genes near L1 insertions. In the case of species 3, the gene that must be silenced is now silenced redundantly by the ancestral mechanism and L1TD1. Because of this redundancy, either *L1TD1* or the ancestral silencing could be lost. D. Loss of the ancestral silencing mechanism would render *L1TD1* essential. A loss of *L1TD1* in this case (species 3) would result in the expression of the orange gene, which must remain silenced for survival. In this way, *L1TD1* could be co-opted as an essential regulator of pluripotency. If L1TD1 instead targets L1s near arbitrary genes with no influence on the pluripotent state of a cell (species 1 and 2), loss of *L1TD1* would not affect the maintenance of pluripotency; in this case, *L1TD1* would be retained only if its restriction or other functions were beneficial.
(PDF)

Table S1 Maximum likelihood estimates of pairwise dN/dS for *L1TD1* from Sigmodontinae rodents.
(XLSX)

Table S2 *L1TD1* Genbank accessions or coordinates in mammalian genome assemblies searched.
(XLSX)

## References

[1] Pardue ML, DeBaryshe PG (2011) Retrotransposons that maintain chromosome ends. Proc Natl Acad Sci USA 108: 20317–20324. 21821789 10.1073/pnas.1100278108 PMC3251079

[2] Cordaux R, Batzer MA (2009) The impact of retrotransposons on human genome evolution. Nat Rev Genet 10: 691–703. 19763152 10.1038/nrg2640 PMC2884099

[3] Brosius J, Gould SJ (1992) On "genomenclature": a comprehensive (and respectful) taxonomy for pseudogenes and other "junk DNA". Proc Natl Acad Sci U S A 89: 10706–10710. 1279691 10.1073/pnas.89.22.10706 PMC50410

[4] Feschotte C (2008) Transposable elements and the evolution of regulatory networks. Nat Rev Genet 9: 397–405. 18368054 10.1038/nrg2337 PMC2596197

[5] Smit AF (1999) Interspersed repeats and other mementos of transposable elements in mammalian genomes. Curr Opin Genet Dev 9: 657–663. 10607616 10.1016/s0959-437x(99)00031-3

[6] Sinzelle L, Izsvak Z, Ivics Z (2009) Molecular domestication of transposable elements: from detrimental parasites to useful host genes. Cell Mol Life Sci 66: 1073–1093. 19132291 10.1007/s00018-009-8376-3 PMC11131479

[7] Mi S, Lee X, Li X, Veldman GM, Finnerty H, et al (2000) Syncytin is a captive retroviral envelope protein involved in human placental morphogenesis. Nature 403: 785–789. 10693809 10.1038/35001608

[8] Black SG, Arnaud F, Palmarini M, Spencer TE (2010) Endogenous retroviruses in trophoblast differentiation and placental development. Am J Reprod Immunol 64: 255–264. 20528833 10.1111/j.1600-0897.2010.00860.x PMC4198168

[9] Dupressoir A, Lavialle C, Heidmann T (2012) From ancestral infectious retroviruses to bona fide cellular genes: role of the captured syncytins in placentation. Placenta 33: 663–671. 22695103 10.1016/j.placenta.2012.05.005

[10] Dupressoir A, Vernochet C, Bawa O, Harper F, Pierron G, et al (2009) Syncytin-A knockout mice demonstrate the critical role in placentation of a fusogenic, endogenous retrovirus-derived, envelope gene. Proc Natl Acad Sci USA 106: 12127–12132. 19564597 10.1073/pnas.0902925106 PMC2715540

[11] Ono R, Nakamura K, Inoue K, Naruse M, Usami T, et al (2006) Deletion of Peg10, an imprinted gene acquired from a retrotransposon, causes early embryonic lethality. Nat Genet 38: 101–106. 16341224 10.1038/ng1699

[12] Suzuki S, Ono R, Narita T, Pask AJ, Shaw G, et al (2007) Retrotransposon silencing by DNA methylation can drive mammalian genomic imprinting. PLoS Genet 3: e55. 17432937 10.1371/journal.pgen.0030055 PMC1851980

[13] Benit L, De Parseval N, Casella JF, Callebaut I, Cordonnier A, et al (1997) Cloning of a new murine endogenous retrovirus, MuERV-L, with strong similarity to the human HERV-L element and with a gag coding sequence closely related to the Fv1 restriction gene. J Virol 71: 5652–5657. 9188643 10.1128/jvi.71.7.5652-5657.1997 PMC191811

[14] Best S, Le Tissier P, Towers G, Stoye JP (1996) Positional cloning of the mouse retrovirus restriction gene Fv1. Nature 382: 826–829. 8752279 10.1038/382826a0

[15] Yan Y, Buckler-White A, Wollenberg K, Kozak CA (2009) Origin, antiviral function and evidence for positive selection of the gammaretrovirus restriction gene Fv1 in the genus Mus. Proc Natl Acad Sci USA 106: 3259–3263. 19221034 10.1073/pnas.0900181106 PMC2651326

[16] Jung YT, Kozak CA (2000) A single amino acid change in the murine leukemia virus capsid gene responsible for the Fv1(nr) phenotype. J Virol 74: 5385–5387. 10799620 10.1128/jvi.74.11.5385-5387.2000 PMC110898

[17] Kozak CA, Chakraborti A (1996) Single amino acid changes in the murine leukemia virus capsid protein gene define the target of Fv1 resistance. Virology 225: 300–305. 8918916 10.1006/viro.1996.0604

[18] Stevens A, Bock M, Ellis S, LeTissier P, Bishop KN, et al (2004) Retroviral capsid determinants of Fv1 NB and NR tropism. J Virol 78: 9592–9598. 15331691 10.1128/JVI.78.18.9592-9598.2004 PMC514981

[19] Kozak CA (1985) Analysis of wild-derived mice for Fv-1 and Fv-2 murine leukemia virus restriction loci: a novel wild mouse Fv-1 allele responsible for lack of host range restriction. J Virol 55: 281–285. 2991555 10.1128/jvi.55.2.281-285.1985 PMC254931

[20] Malik HS, Burke WD, Eickbush TH (1999) The age and evolution of non-LTR retrotransposable elements. Mol Biol Evol 16: 793–805. 10368957 10.1093/oxfordjournals.molbev.a026164

[21] Brosius J (1999) RNAs from all categories generate retrosequences that may be exapted as novel genes or regulatory elements. Gene 238: 115–134. 10570990 10.1016/s0378-1119(99)00227-9

[22] Feschotte C, Pritham EJ (2007) DNA transposons and the evolution of eukaryotic genomes. Annu Rev Genet 41: 331–368. 18076328 10.1146/annurev.genet.40.110405.090448 PMC2167627

[23] Volff JN (2006) Turning junk into gold: domestication of transposable elements and the creation of new genes in eukaryotes. Bioessays 28: 913–922. 16937363 10.1002/bies.20452

[24] Kuwabara T, Hsieh J, Muotri A, Yeo G, Warashina M, et al (2009) Wnt-mediated activation of NeuroD1 and retro-elements during adult neurogenesis. Nat Neurosci 12: 1097–1105. 19701198 10.1038/nn.2360 PMC2764260

[25] Lev-Maor G, Sorek R, Shomron N, Ast G (2003) The birth of an alternatively spliced exon: 3' splice-site selection in Alu exons. Science 300: 1288–1291. 12764196 10.1126/science.1082588

[26] Sorek R, Ast G, Graur D (2002) Alu-containing exons are alternatively spliced. Genome Res 12: 1060–1067. 12097342 10.1101/gr.229302 PMC186627

[27] Lorenc A, Makalowski W (2003) Transposable elements and vertebrate protein diversity. Genetica 118: 183–191. 12868608

[28] Sela N, Kim E, Ast G (2010) The role of transposable elements in the evolution of non-mammalian vertebrates and invertebrates. Genome Biol 11: R59. 20525173 10.1186/gb-2010-11-6-r59 PMC2911107

[29] Iwashita S, Ueno S, Nakashima K, Song SY, Ohshima K, et al (2006) A tandem gene duplication followed by recruitment of a retrotransposon created the paralogous bucentaur gene (bcntp97) in the ancestral ruminant. Mol Biol Evol 23: 798–806. 16384818 10.1093/molbev/msj088

[30] Mitsui K, Tokuzawa Y, Itoh H, Segawa K, Murakami M, et al (2003) The homeoprotein Nanog is required for maintenance of pluripotency in mouse epiblast and ES cells. Cell 113: 631–642. 12787504 10.1016/s0092-8674(03)00393-3

[31] Wong RC, Ibrahim A, Fong H, Thompson N, Lock LF, et al (2011) L1TD1 is a marker for undifferentiated human embryonic stem cells. PLoS ONE 6: e19355. 21559406 10.1371/journal.pone.0019355 PMC3084827

[32] Narva E, Rahkonen N, Emani MR, Lund R, Pursiheimo JP, et al (2012) RNA-binding protein L1TD1 interacts with LIN28 via RNA and is required for human embryonic stem cell self-renewal and cancer cell proliferation. Stem Cells 30: 452–460. 22162396 10.1002/stem.1013 PMC3507993

[33] Iwabuchi KA, Yamakawa T, Sato Y, Ichisaka T, Takahashi K, et al (2011) ECAT11/L1td1 is enriched in ESCs and rapidly activated during iPSC generation, but it is dispensable for the maintenance and induction of pluripotency. PLoS ONE 6: e20461. 21637830 10.1371/journal.pone.0020461 PMC3102727

[34] Martin SL (2006) The ORF1 protein encoded by LINE-1: structure and function during L1 retrotransposition. Journal of biomedicine & biotechnology 2006: 45621. 16877816 10.1155/JBB/2006/45621 PMC1510943

[35] Wei W, Gilbert N, Ooi SL, Lawler JF, Ostertag EM, et al (2001) Human L1 retrotransposition: cis preference versus trans complementation. Mol Cell Biol 21: 1429–1439. 11158327 10.1128/MCB.21.4.1429-1439.2001 PMC99594

[36] Kulpa DA, Moran JV (2006) Cis-preferential LINE-1 reverse transcriptase activity in ribonucleoprotein particles. Nat Struct Mol Biol 13: 655–660. 16783376 10.1038/nsmb1107

[37] Daugherty MD, Malik HS (2012) Rules of engagement: molecular insights from host-virus arms races. Annu Rev Genet 46: 677–700. 23145935 10.1146/annurev-genet-110711-155522

[38] Khan H, Smit A, Boissinot S (2006) Molecular evolution and tempo of amplification of human LINE-1 retrotransposons since the origin of primates. Genome Res 16: 78–87. 16344559 10.1101/gr.4001406 PMC1356131

[39] Nielsen R, Yang Z (1998) Likelihood models for detecting positively selected amino acid sites and applications to the HIV-1 envelope gene. Genetics 148: 929–936. 9539414 10.1093/genetics/148.3.929 PMC1460041

[40] Boissinot S, Furano AV (2001) Adaptive evolution in LINE-1 retrotransposons. Mol Biol Evol 18: 2186–2194. 11719568 10.1093/oxfordjournals.molbev.a003765

[41] Kosakovsky Pond SL, Posada D, Gravenor MB, Woelk CH, Frost SD (2006) Automated phylogenetic detection of recombination using a genetic algorithm. Mol Biol Evol 23: 1891–1901. 16818476 10.1093/molbev/msl051

[42] Gibbs RA, Rogers J, Katze MG, Bumgarner R, Weinstock GM, et al (2007) Evolutionary and biomedical insights from the rhesus macaque genome. Science 316: 222–234. 17431167 10.1126/science.1139247

[43] Chia NY, Chan YS, Feng B, Lu X, Orlov YL, et al (2010) A genome-wide RNAi screen reveals determinants of human embryonic stem cell identity. Nature 468: 316–320. 20953172 10.1038/nature09531

[44] Cantrell MA, Scott L, Brown CJ, Martinez AR, Wichman HA (2008) Loss of LINE-1 activity in the megabats. Genetics 178: 393–404. 18202382 10.1534/genetics.107.080275 PMC2206088

[45] Grahn RA, Rinehart TA, Cantrell MA, Wichman HA (2005) Extinction of LINE-1 activity coincident with a major mammalian radiation in rodents. Cytogenet Genome Res 110: 407–415. 16093693 10.1159/000084973

[46] Cantrell MA, Ederer MM, Erickson IK, Swier VJ, Baker RJ, et al (2005) MysTR: an endogenous retrovirus family in mammals that is undergoing recent amplifications to unprecedented copy numbers. J Virol 79: 14698–14707. 16282470 10.1128/JVI.79.23.14698-14707.2005 PMC1287555

[47] Erickson IK, Cantrell MA, Scott L, Wichman HA (2011) Retrofitting the genome: L1 extinction follows endogenous retroviral expansion in a group of muroid rodents. J Virol 85: 12315–12323. 21957310 10.1128/JVI.05180-11 PMC3209412

[48] Smit AF, Hubley R, Green P (1996–2004) RepeatMasker Open-3.0. http://www.repeatmasker.org.

[49] Lander ES, Linton LM, Birren B, Nusbaum C, Zody MC, et al (2001) Initial sequencing and analysis of the human genome. Nature 409: 860–921. 11237011 10.1038/35057062

[50] Ono R, Shiura H, Aburatani H, Kohda T, Kaneko-Ishino T, et al (2003) Identification of a large novel imprinted gene cluster on mouse proximal chromosome 6. Genome Res 13: 1696–1705. 12840045 10.1101/gr.906803 PMC403743

[51] Esnault C, Cornelis G, Heidmann O, Heidmann T (2013) Differential evolutionary fate of an ancestral primate endogenous retrovirus envelope gene, the EnvV syncytin, captured for a function in placentation. PLoS Genet 9: e1003400. 23555306 10.1371/journal.pgen.1003400 PMC3610889

[52] Zamudio N, Bourc'his D (2010) Transposable elements in the mammalian germline: a comfortable niche or a deadly trap? Heredity (Edinb) 105: 92–104. 20442734 10.1038/hdy.2010.53

[53] Dewannieux M, Esnault C, Heidmann T (2003) LINE-mediated retrotransposition of marked Alu sequences. Nat Genet 35: 41–48. 12897783 10.1038/ng1223

[54] Bao W, Jurka J (2010) Origin and evolution of LINE-1 derived "half-L1" retrotransposons (HAL1). Gene 465: 9–16. 20600705 10.1016/j.gene.2010.06.005 PMC2923044

[55] Khazina E, Truffault V, Buttner R, Schmidt S, Coles M, et al (2011) Trimeric structure and flexibility of the L1ORF1 protein in human L1 retrotransposition. Nat Struct Mol Biol 18: 1006–1014. 21822284 10.1038/nsmb.2097

[56] Wolf D, Goff SP (2009) Embryonic stem cells use ZFP809 to silence retroviral DNAs. Nature 458: 1201–1204. 19270682 10.1038/nature07844 PMC2676211

[57] Rowe HM, Trono D (2011) Dynamic control of endogenous retroviruses during development. Virology 411: 273–287. 21251689 10.1016/j.virol.2010.12.007

[58] Rebollo R, Farivar S, Mager DL (2012) C-GATE - catalogue of genes affected by transposable elements. Mobile DNA 3: 9. 22621612 10.1186/1759-8753-3-9 PMC3472293

[59] Rebollo R, Romanish MT, Mager DL (2012) Transposable elements: an abundant and natural source of regulatory sequences for host genes. Annu Rev Genet 46: 21–42. 22905872 10.1146/annurev-genet-110711-155621

[60] Jordan IK, Rogozin IB, Glazko GV, Koonin EV (2003) Origin of a substantial fraction of human regulatory sequences from transposable elements. Trends Genet 19: 68–72. 12547512 10.1016/s0168-9525(02)00006-9

[61] Szak ST, Pickeral OK, Makalowski W, Boguski MS, Landsman D, et al (2002) Molecular archeology of L1 insertions in the human genome. Genome Biol 3: R52. 10.1186/gb-2002-3-10-research0052 PMC134481 12372140

[62] Lohse M, Bolger AM, Nagel A, Fernie AR, Lunn JE, et al (2012) RobiNA: a user-friendly, integrated software solution for RNA-Seq-based transcriptomics. Nucleic Acids Res 40: W622–627. 22684630 10.1093/nar/gks540 PMC3394330

[63] Fujita PA, Rhead B, Zweig AS, Hinrichs AS, Karolchik D, et al (2011) The UCSC Genome Browser database: update 2011. Nucleic Acids Res 39: D876–882. 20959295 10.1093/nar/gkq963 PMC3242726

[64] Sedlazeck FJ, Rescheneder P, von Haeseler A (2013) NextGenMap: fast and accurate read mapping in highly polymorphic genomes. Bioinformatics 29: 2790–2791. 23975764 10.1093/bioinformatics/btt468

[65] Haas BJ, Papanicolaou A, Yassour M, Grabherr M, Blood PD, et al (2013) De novo transcript sequence reconstruction from RNA-seq using the Trinity platform for reference generation and analysis. Nat Protoc 8: 1494–1512. 23845962 10.1038/nprot.2013.084 PMC3875132

[66] Altschul SF, Madden TL, Schaffer AA, Zhang J, Zhang Z, et al (1997) Gapped BLAST and PSI-BLAST: a new generation of protein database search programs. Nucleic Acids Res 25: 3389–3402. 9254694 10.1093/nar/25.17.3389 PMC146917

[67] Li H, Handsaker B, Wysoker A, Fennell T, Ruan J, et al (2009) The Sequence Alignment/Map format and SAMtools. Bioinformatics 25: 2078–2079. 19505943 10.1093/bioinformatics/btp352 PMC2723002

[68] McGinnis S, Madden TL (2004) BLAST: at the core of a powerful and diverse set of sequence analysis tools. Nucleic Acids Res 32: W20–25. 15215342 10.1093/nar/gkh435 PMC441573

[69] Perelman P, Johnson WE, Roos C, Seuanez HN, Horvath JE, et al (2011) A molecular phylogeny of living primates. PLoS Genet 7: e1001342. 21436896 10.1371/journal.pgen.1001342 PMC3060065

[70] Yang Z (1997) PAML: a program package for phylogenetic analysis by maximum likelihood. Comput Appl Biosci 13: 555–556. 9367129 10.1093/bioinformatics/13.5.555

[71] Kosakovsky Pond SL, Frost SDW (2005) Not So Different After All: A Comparison of Methods for Detecting Amino Acid Sites Under Selection. Mol Biol Evol 22: 1208–1222. 15703242 10.1093/molbev/msi105

[72] Murrell B, Moola S, Mabona A, Weighill T, Sheward D, et al (2013) FUBAR: a fast, unconstrained bayesian approximation for inferring selection. Mol Biol Evol 30: 1196–1205. 23420840 10.1093/molbev/mst030 PMC3670733

[73] Gentleman RC, Carey VJ, Bates DM, Bolstad B, Dettling M, et al (2004) Bioconductor: open software development for computational biology and bioinformatics. Genome Biol 5: R80. 15461798 10.1186/gb-2004-5-10-r80 PMC545600

[74] Sonnhammer EL, Durbin R (1995) A dot-matrix program with dynamic threshold control suited for genomic DNA and protein sequence analysis. Gene 167: GC1–10. 8566757 10.1016/0378-1119(95)00714-8

[75] Larkin MA, Blackshields G, Brown NP, Chenna R, McGettigan PA, et al (2007) Clustal W and Clustal X version 2.0. Bioinformatics 23: 2947–2948. 17846036 10.1093/bioinformatics/btm404

[76] Jurka J, Kapitonov VV, Pavlicek A, Klonowski P, Kohany O, et al (2005) Repbase Update, a database of eukaryotic repetitive elements. Cytogenet Genome Res 110: 462–467. 16093699 10.1159/000084979

[77] Darriba D, Taboada GL, Doallo R, Posada D (2012) jModelTest 2: more models, new heuristics and parallel computing. Nat Methods 9: 772. 22847109 10.1038/nmeth.2109 PMC4594756

[78] Guindon S, Gascuel O (2003) A simple, fast, and accurate algorithm to estimate large phylogenies by maximum likelihood. Syst Biol 52: 696–704. 14530136 10.1080/10635150390235520

[79] Tamura K, Peterson D, Peterson N, Stecher G, Nei M, et al (2011) MEGA5: molecular evolutionary genetics analysis using maximum likelihood, evolutionary distance, and maximum parsimony methods. Mol Biol Evol 28: 2731–2739. 21546353 10.1093/molbev/msr121 PMC3203626

[80] Darriba D, Taboada GL, Doallo R, Posada D (2011) ProtTest 3: fast selection of best-fit models of protein evolution. Bioinformatics 27: 1164–1165. 21335321 10.1093/bioinformatics/btr088 PMC5215816

[81] Kumar S, Hedges SB (2011) TimeTree2: species divergence times on the iPhone. Bioinformatics 27: 2023–2024. 21622662 10.1093/bioinformatics/btr315 PMC3129528

[82] Almeida FC, Giannini NP, DeSalle R, Simmons NB (2011) Evolutionary relationships of the old world fruit bats (Chiroptera, Pteropodidae): another star phylogeny? BMC Evol Biol 11: 281. 21961908 10.1186/1471-2148-11-281 PMC3199269

