# Supplementary figures and images for: Positive Selection and Multiple Losses of the LINE-1-Derived L1TD1 Gene in Mammals Suggest a Dual Role in Genome Defense and Pluripotency
Source: PLoS Genet. 2014 Sep 11;10(9):e1004531. doi: 10.1371/journal.pgen.1004531 (PMC4161310; doi:10.1371/journal.pgen.1004531)

Figure S1

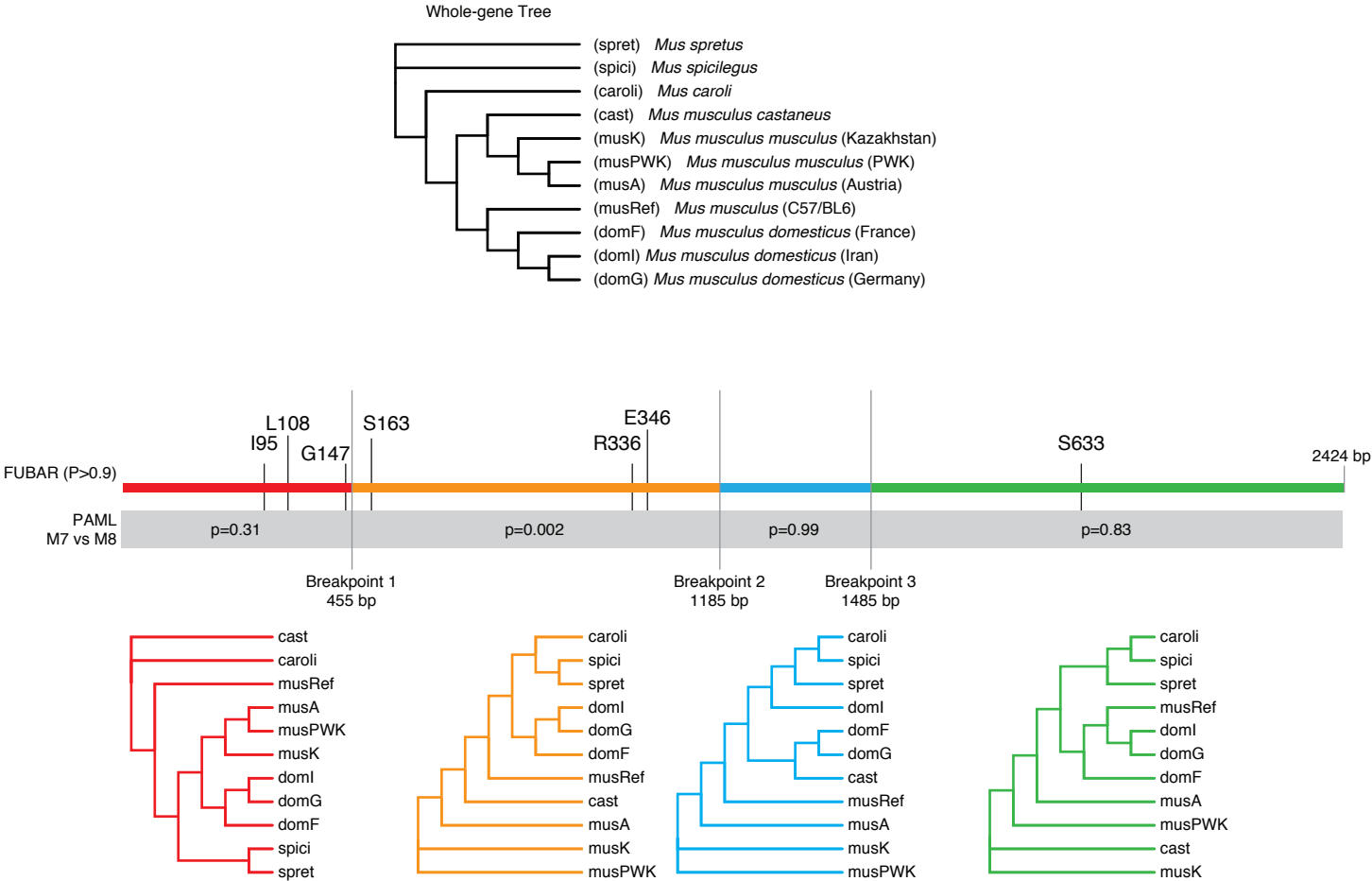

Supplement: Figure S1 — Positive selection in Mus L1TD1 is robust to alternative phylogenies from potential recombination breakpoints. We used HyPhy's GARD program to test for recombination breakpoints in the Mus L1TD1 alignment that could give rise to false signatures of positive selection. We found three potential breakpoints, though none was statistically significant (KH test, p>0.1). To ensure that our detection of positive selection was robust to the use of these alternative phylogenies, we performed PAML NSsites on slices of the alignment corresponding to each of the GARD trees, and we used the built-in functionality of DataMonkey to use these GARD-generated trees to identify positively-selected positions using FUBAR. Top, the whole gene tree is shown in black. The three breakpoints are shown on the schematic of the L1TD1 gene with vertical lines, and the slices they delimit are shown in different colors. Below each gene segment is shown the GARD-generated tree that best describes that region. Both the PAML NSsites signature of selection and FUBAR-identified selected sites are robust to the use of these alternative phylogenies. (PDF) [file pgen.1004531.s003.pdf]

Figure S2

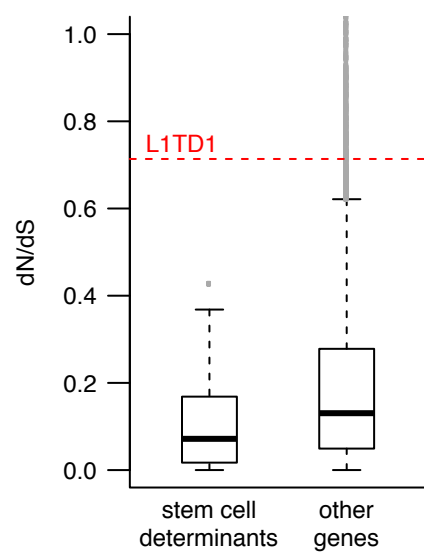

Supplement: Figure S2 — Determinants of embryonic stem cell identity evolve more slowly than control genes. The boxplots show the distribution of dN/dS values for genes identified in a screen for determinants of human ES cell identity [43] and for all other genes in a genome-wide dataset of dN/dS values calculated from trios of orthologs from human, chimpanzee and macaque genomes [42]. Outliers with dN/dS>1 are omitted; gray dots represent other outliers - the large number of datapoints for "other genes" precludes visualization of individual datapoints. The stem cell determinants are evolving more slowly than other genes (Wilcoxon p = 0.008). L1TD1's dN/dS value in this genome-wide dataset is shown using a red horizontal line; it is evolving faster than most other pluripotency genes. Although L1TD1 did not meet the arbitrary threshold (Fav score <−2) used to identify stem cell determinants in the published RNAi screen, its score in the screen (Fav = −0.90) is well below the genome-wide average, consistent with previous results that human L1TD1 is a pluripotency factor [31], [32]. (PDF) [file pgen.1004531.s004.pdf]

Figure S3

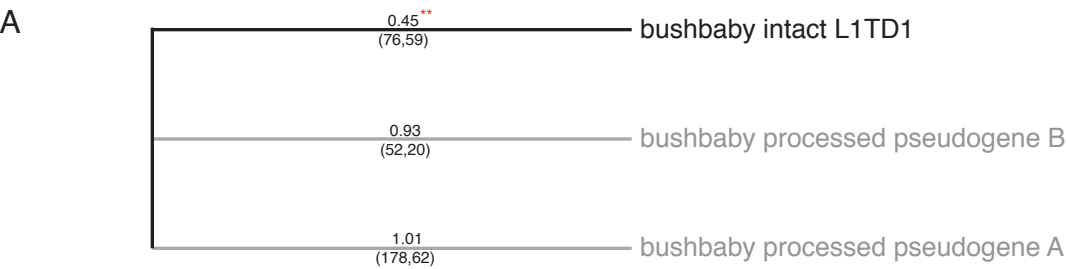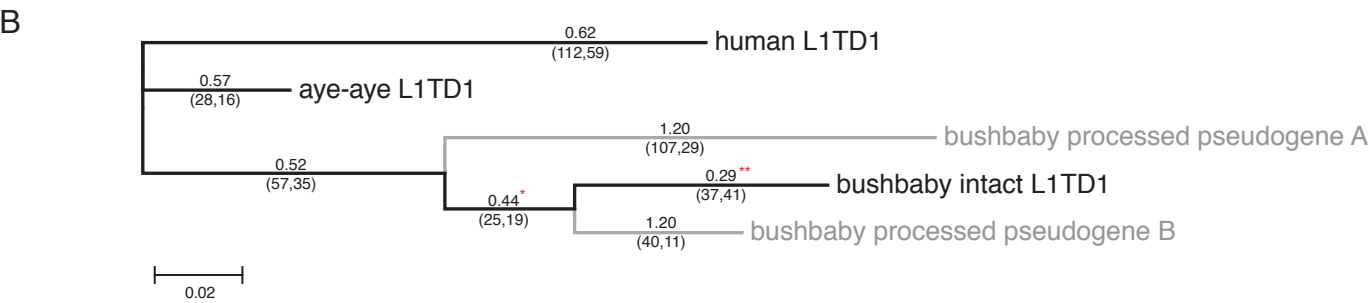

Supplement: Figure S3 — Intact L1TD1 from bushbaby Otolemur garnettii has evolved under purifying selection. We used codeml's free-ratio model to estimate selective pressures on L1TD1 on each branch of the two trees shown. Above each branch we show estimated dN/dS ratios, and in parentheses below each branch we show the estimated number of non-synonymous and synonymous changes, respectively. L1TD1 pseudogenes are shown with their labels in gray. For three selected branches we performed likelihood tests of whether the estimated dN/dS ratio is a significantly better fit to the data than dN/dS = 1 for that branch, by assuming only two dN/dS ratios for the entire tree (one ratio for the branch in question, and one for all other branches), and comparing a model where dN/dS for the branch in question was fixed at 1 with a model where dN/dS was freely estimated. Results of those tests are shown with red superscripts. A. We examined evolution of the full-length ORF of the novel bushbaby L1TD1 structure, comparing it to two processed pseudogenes in the bushbaby genome that arose after this novel L1TD1 structure formed. The intact bushbaby L1TD1 is more likely evolving under purifying than neutral selection (p = 0.0003; **). B. We examined the portion of bushbaby L1TD1 that aligns to the ancestral L1TD1 gene, including human and aye-aye L1TD1 genes as outgroups. The intact bushbaby L1TD1 is more likely evolving under purifying than neutral selection (p<10−5; **); there is also weaker support (p = 0.07; *) for purifying selection on the shared ancestor of the intact bushbaby L1TD1 and bushbaby processed pseudogene B. (PDF) [file pgen.1004531.s005.pdf]

Figure S4

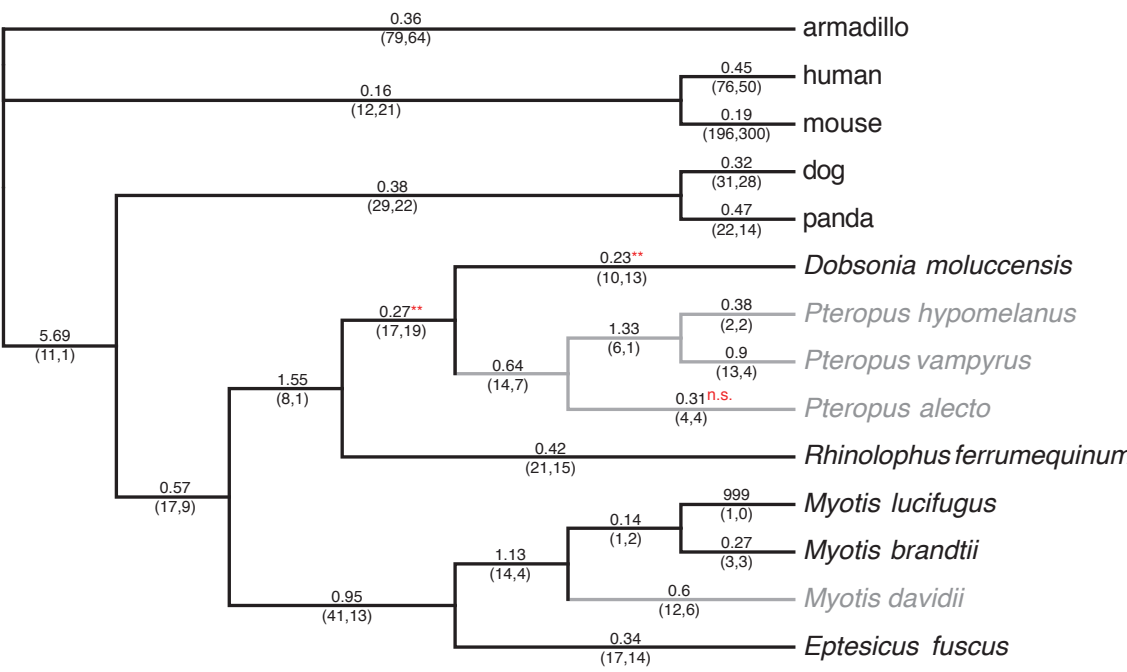

Supplement: Figure S4 — Intact L1TD1 from megabat Dobsonia moluccensis has evolved under purifying selection. We used codeml's free-ratio model to estimate selective pressures on L1TD1 on each branch of the species tree shown. Above each branch we show estimated dN/dS ratios, and in parentheses below each branch we show the estimated number of non-synonymous and synonymous changes, respectively. Species in which L1TD1 is a pseudogene are shown with their labels in gray. For three branches, we performed likelihood tests of whether the estimated dN/dS ratio is a significantly better fit to the data than dN/dS = 1 for that branch, by assuming only two dN/dS ratios for the entire tree (one ratio for the branch in question, and one for all other branches), and comparing a model where dN/dS for the branch in question was fixed at 1 with a model where dN/dS was freely estimated. Results of those tests are shown with red superscripts: the Dobsonia moluccensis branch is more likely evolving under purifying than neutral selection (p = 0.003), as is the branch ancestral to D. moluccensis and the three Pteropus species (p = 0.002). In contrast, for the Pteropus alecto branch, a neutral model is as good a fit to the data as a model invoking purifying selection (denoted by n.s., for non-significant). (PDF) [file pgen.1004531.s006.pdf]

Figure S5

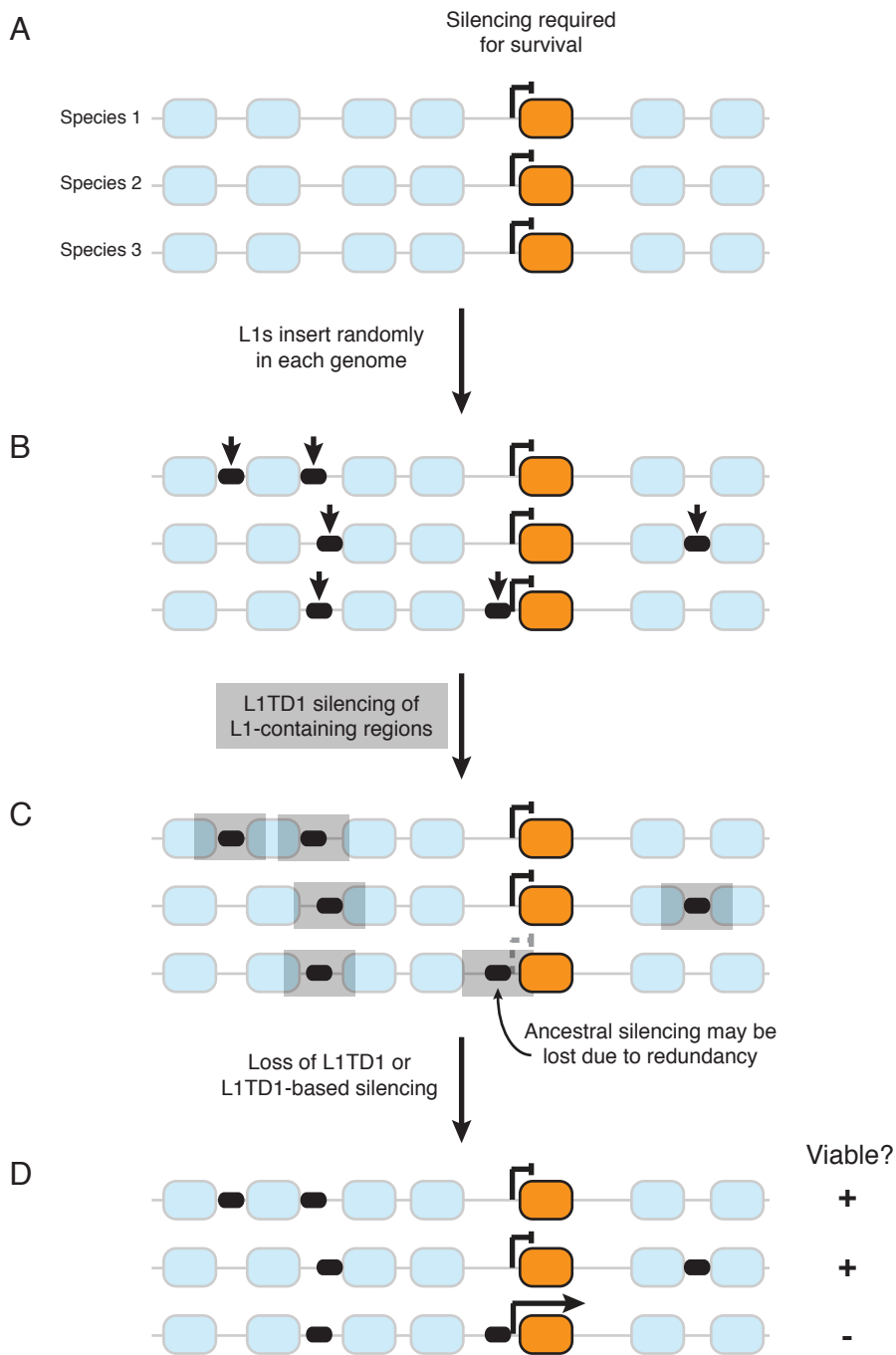

Supplement: Figure S5 — L1TD1 could become essential based upon the pattern of L1 insertions in a specific genome. We present a scheme under which L1TD1 could idiosyncratically adopt a pluripotency role in a species-specific fashion. A. We schematize the genomes of three exemplar species with an identical stretch of seven genes (rounded boxes), one of which must be silenced for pluripotency maintenance (orange boxes). B. L1s (black rounded boxes) insert randomly into each genome. One insertion (species 3) happens to be near the silenced gene. C. According to our hypothesis, L1TD1 silences the newly inserted L1s (grayed areas), as well as genes near L1 insertions. In the case of species 3, the gene that must be silenced is now silenced redundantly by the ancestral mechanism and L1TD1. Because of this redundancy, either L1TD1 or the ancestral silencing could be lost. D. Loss of the ancestral silencing mechanism would render L1TD1 essential. A loss of L1TD1 in this case (species 3) would result in the expression of the orange gene, which must remain silenced for survival. In this way, L1TD1 could be co-opted as an essential regulator of pluripotency. If L1TD1 instead targets L1s near arbitrary genes with no influence on the pluripotent state of a cell (species 1 and 2), loss of L1TD1 would not affect the maintenance of pluripotency; in this case, L1TD1 would be retained only if its restriction or other functions were beneficial. (PDF) [file pgen.1004531.s007.pdf]
